# Deletion of IL-4 Receptor Alpha on Dendritic Cells Renders BALB/c Mice Hypersusceptible to *Leishmania major* Infection

**DOI:** 10.1371/journal.ppat.1003699

**Published:** 2013-10-24

**Authors:** Ramona Hurdayal, Natalie E. Nieuwenhuizen, Mélanie Revaz-Breton, Liezel Smith, Jennifer C. Hoving, Suraj P. Parihar, Boris Reizis, Frank Brombacher

**Affiliations:** 1 International Center for Genetic Engineering and Biotechnology (ICGEB), Cape Town Component and Institute of Infectious Diseases and Molecular Medicine (IIDMM), Division of Immunology, University of Cape Town, Cape Town, South Africa; 2 Max Planck Institute for Infection Biology, Department Immunology, Berlin, Germany; 3 Lung Infection and Immunity Unit, Department of Medicine, Groote Schuur Hospital, University of Cape Town, Cape Town, South Africa; 4 Columbia University Medical Center, Department of Microbiology, New York, New York, United States of America; Imperial College London, United Kingdom

## Abstract

In BALB/c mice, susceptibility to infection with the intracellular parasite *Leishmania major* is driven largely by the development of T helper 2 (Th2) responses and the production of interleukin (IL)-4 and IL-13, which share a common receptor subunit, the IL-4 receptor alpha chain (IL-4Rα). While IL-4 is the main inducer of Th2 responses, paradoxically, it has been shown that exogenously administered IL-4 can promote dendritic cell (DC) IL-12 production and enhance Th1 development if given early during infection. To further investigate the relevance of biological quantities of IL-4 acting on DCs during *in vivo* infection, DC specific IL-4Rα deficient (CD11c^cre^IL-4Rα^-/lox^) BALB/c mice were generated by gene targeting and site-specific recombination using the cre/*lox*P system under control of the *cd11c* locus. DNA, protein, and functional characterization showed abrogated IL-4Rα expression on dendritic cells and alveolar macrophages in CD11c^cre^IL-4Rα^-/lox^ mice. Following infection with *L. major*, CD11c^cre^IL-4Rα^-/lox^ mice became hypersusceptible to disease, presenting earlier and increased footpad swelling, necrosis and parasite burdens, upregulated Th2 cytokine responses and increased type 2 antibody production as well as impaired classical activation of macrophages. Hypersusceptibility in CD11c^cre^IL-4Rα^-/lox^ mice was accompanied by a striking increase in parasite burdens in peripheral organs such as the spleen, liver, and even the brain. DCs showed increased parasite loads in CD11c^cre^IL-4Rα^-/lox^ mice and reduced iNOS production. IL-4Rα-deficient DCs produced reduced IL-12 but increased IL-10 due to impaired DC instruction, with increased mRNA expression of IL-23p19 and activin A, cytokines previously implicated in promoting Th2 responses. Together, these data demonstrate that abrogation of IL-4Rα signaling on DCs is severely detrimental to the host, leading to rapid disease progression, and increased survival of parasites in infected DCs due to reduced killing effector functions.

## Author Summary

Leishmaniasis is a parasitic infection caused by protozoan parasites of *Leishmania* species and is transmitted by the sandfly. Disease in humans ranges from localized cutaneous lesions to disseminated visceral Leishmaniasis. Mouse models of *Leishmania major* infection have demonstrated that a “healing” response in C57BL/6 mice requires the secretion of protective T helper (Th) 1 cytokines, including IFN-γ, which mediates parasite killing by inducing nitric oxide production. Conversely, “non-healer” BALB/c mice are unable to control infection and develop a Th2 immune response characterized by the production of IL-4 and IL-13 cytokines. Although IL-4 is the main inducer of Th2 responses, it has been shown that IL-4 can instruct dendritic cell (DC)-derived IL-12 production and Th1 development if administered during DC activation. To further investigate the role of DCs, a DC specific IL-4Rα-deficient mouse model was established. *L. major* studies demonstrated hypersusceptibility to infection and strikingly increased parasite loads in peripheral organs of mice lacking IL-4Rα on DCs. Moreover, increased parasite burdens were observed in host cells, including DCs, which showed reduced killing effector functions. In summary, this study demonstrates that IL-4Rα-mediated instruction of DCs occurs *in vivo* and is necessary to avoid rapid progression of disease in the host.

## Introduction

*Leishmania* spp. are protozoan parasites that are transmitted by *Phlebotomus* spp. sandflies and can cause several forms of disease in humans, ranging from localized cutaneous lesions to visceral Leishmaniasis, where parasites invade internal organs such as the spleen and liver. The incidence of disease is approximately 1.5 million per annum for cutaneous Leishmaniasis, and 500 000 per annum for visceral Leishmaniasis, which is usually fatal if left untreated [1]. Currently there is no vaccine. To identify correlates of immune protection, which may aid in vaccine design and therapeutic strategies, experimental models of cutaneous Leishmaniasis have been established in which disease is induced by infecting mice subcutaneously with *L. major*. Susceptible BALB/c mice show progressive lesion development with dissemination of parasites to visceral organs, while resistant C57BL/6 mice are able to control infection and heal lesions [2]–[4]. Lack of healing in BALB/c mice is associated with a T helper (Th) 2 response characterized by secretion of interleukin (IL)-4, IL-5, IL-9 and IL-13 [3], [5]–[8], high anti-*Leishmania* antibody titres [8], [9] and alternative activation of macrophages [9], [10]. In contrast, resistant C57BL/6 mice develop protective Th1 responses with production of IL-12 and IFN-γ, associated with classical activation of macrophages and killing of parasites by effector nitric oxide production [9], [11]–[14]. IL-4 and IL-13, both of which signal through a common receptor chain, the IL-4 receptor alpha (IL-4Rα) are known to be important susceptibility factors in *L. major* infection [3], [6], [8], [15], [16]. Both BALB/c and C57BL/6 mice secrete IL-4 early after infection however, production of IL-4 is sustained in susceptible BALB/c mice and transient in resistant C57BL/6 mice [17], [18]. It appears that resistant mouse strains redirect the early Th2 response in an IL-12-dependent mechanism, while in susceptible mice the Th2 response persists and dominates the disease outcome by suppressing effector mechanisms needed for parasite killing [3].

While IL-4 is the primary inducer of Th2 responses [19], paradoxically it has also been shown that IL-4 promotes IL-12 production by bone marrow-derived dendritic cells (BMDCs) stimulated with CpG or LPS [20]–[23]. Furthermore, administration of 1 µg of recombinant IL-4 at 0 and 8 hours after infection with *L. major* led to increased IL-12 mRNA expression by dendritic cells (DCs) *in vivo*, promoted Th1 responses and rendered mice resistant to infection [21]. It has also been shown that global abrogation of IL-4Rα renders mice resistant to *L. major* only in the acute phase of infection, with mice continuing to develop necrotic footpad lesions during the chronic phase [15]. However, specific abrogation of IL-4Rα on CD4^+^ T cells does lead to resistance, indicating a protective role for IL-4Rα signalling on non-CD4^+^ T cells [24].

A candidate for this protective role may therefore be DCs. These sentinels of the immune system are specialized antigen-presenting cells, proficient at uptake of antigen, migration to the lymph nodes (LN) and activation of lymphocytes. Consequently, they play a critical role in the initiation and differentiation of the adaptive immune response [25], [26]. To investigate the role of IL-4Rα signaling on DCs in resistance to *Leishmania*, CD11c^cre^IL-4Rα^-/lox^ mice, deficient in IL-4Rα signaling on DCs, were generated and infected with *L. major* LV39 and IL81 strains. CD11c^cre^IL-4Rα^-/lox^ mice were hypersusceptible to both strains of *L. major*, with increased footpad swelling and necrosis and substantially increased parasite burdens in peripheral organs, including the brain. Hypersusceptibility in CD11c^cre^IL-4Rα^-/lox^ mice was associated with an upregulation of Th2 responses, impairment in iNOS production by macrophages and inflammatory DCs and increased parasite loads in LN and spleen DCs. Therefore, it is clear that IL-4Rα signaling has important effects on DC phenotype during cutaneous *L. major* infection, and is necessary to avoid rapid disease progression in the host. This study therefore expands our knowledge on the role of dendritic cells during cutaneous Leishmaniasis and on the effects of IL-4Rα signaling on dendritic cells.

## Results

### Generation and characterization of CD11c^cre^IL-4Rα^-/lox^ mice

**Figure 1:**
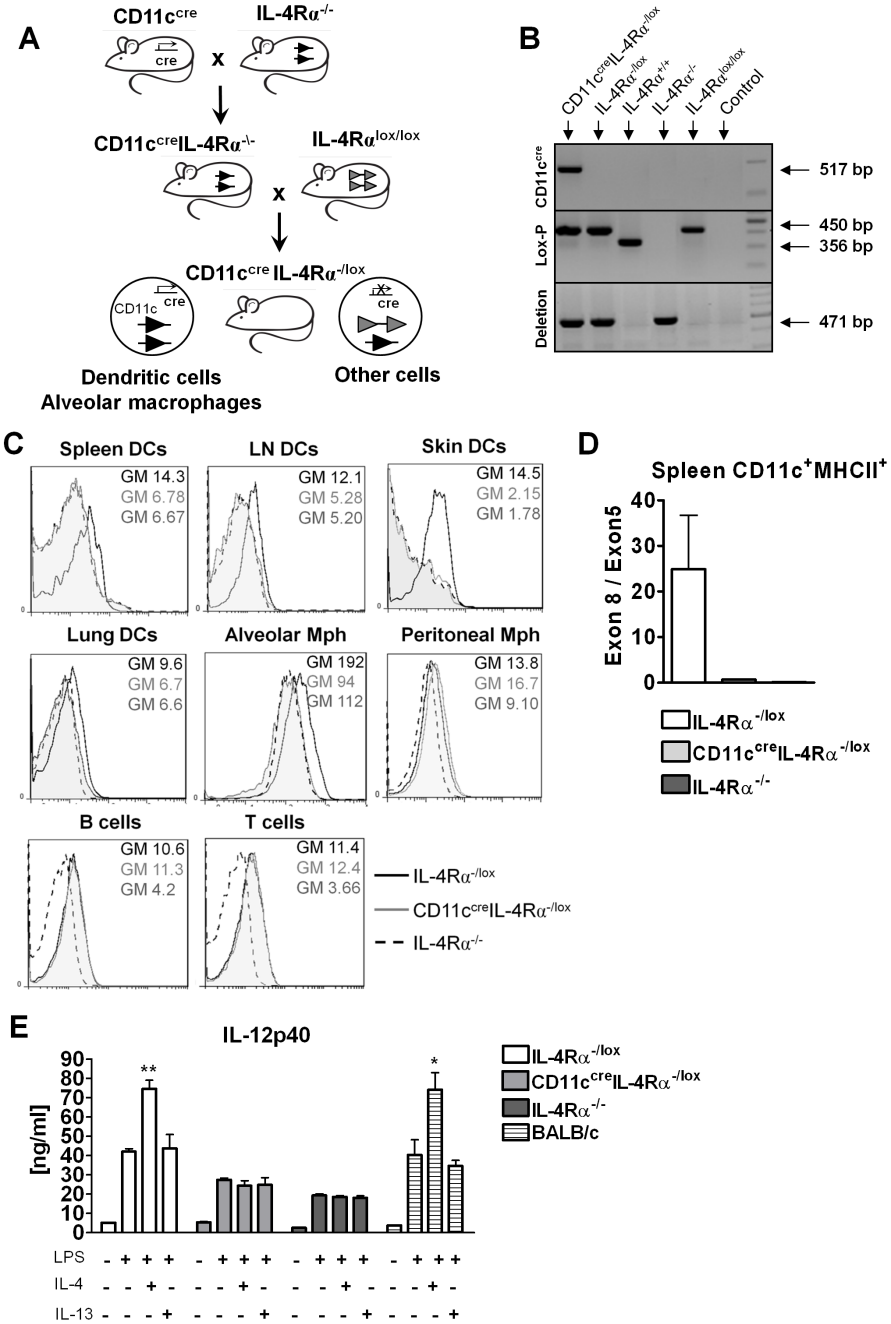
Generation and characterization of CD11c^cre^IL-4Rα^-/lox^ BALB/c mice. (A) IL-4Rα^-/-^ BALB/c mice were intercrossed with CD11c^cre^ expressing and IL-4Rα^lox/lox^ mice to generate CD11c^cre^IL-4Rα^-/lox^ BALB/c mice. (B) Genotyping of CD11c^cre^IL-4Rα^-/lox^ mice. The deleted IL-4Rα PCR is 471 base pairs, loxP is 450 base pairs (floxed) or 356 base pairs (wildtype) and CD11c^cre^ specific is 517 base pairs. (C) IL-4Rα surface expression was analyzed by flow cytometry from naïve IL-4Rα^-/lox^ (solid line), IL-4Rα^-/-^ (dashed line) and CD11c^cre^IL-4Rα^-/lox^ (grey tinted) mice. DCs were CD11c^+^MHCII^+^ (SiglecF^−^ in lungs), alveolar macrophages were CD11c^+^SiglecF^+^, peritoneal macrophages were F480^+^CD11b^+^, B cells were CD19^+^CD3^−^ and T cells were CD3^+^CD19^−^. GM = geometric mean. (D) Genomic DNA was extracted from spleen DCs and IL-4Rα exon 8 (deleted in IL-4Rα deficient cells) was determined by RT-PCR and normalized to exon 5 (present in all cells). (E) Bone marrow-derived DCs were stimulated with LPS in the presence of absence of IL-4 or IL-13 and IL-12p40 was measured in the supernatants 48 hours later. (*, p<0.05, **, *p*≤0.01.

Mice expressing cyclization recombinase (Cre) under control of the *cd11c* locus [27] were backcrossed to BALB/c for 9 generations, then intercrossed with global IL-4Rα (IL-4Rα^-/-^) [15] BALB/c mice to generate CD11c^cre^IL-4Rα^-/-^ BALB/c mice. These mice were subsequently intercrossed with floxed IL-4Rα (IL-4Rα^lox/lox^) BALB/c mice (exon 6 to 8 flanked by *loxP*) [28] to generate CD11c^cre^IL-4Rα^-/lox^ BALB/c mice (Figure 1A). CD11c^cre^IL-4Rα^-/lox^ mice were identified by PCR genotyping (Figure 1B). Analysis of IL-4Rα surface expression on different cell types by flow cytometry demonstrated that IL-4Rα was efficiently depleted in DCs of the lymph nodes, spleen, skin and lungs, when compared with IL-4Rα^-/lox^ littermate controls and IL-4Rα^-/-^ mice (Figure 1C). As expected CD11c^+^ alveolar macrophages also had abrogated IL-4Rα surface expression. Other cell types such as T cells, B cells and macrophages had comparable IL-4Rα expression to IL-4Rα^-/lox^ littermate controls. Cre-mediated IL-4Rα deletion in DCs was confirmed at the genomic level by performing PCR for IL-4Rα exon 8 (absent in IL-4Rα-deficient cells) normalized to IL-4Rα exon 5 (present in all cells) using DNA from CD11c^+^MHCII^+^ DCs sorted from the spleens of naïve mice (Figure 1D).

To assess functional impairment of DCs in CD11c^cre^IL-4Rα^-/lox^ mice, we generated bone marrow-derived dendritic cells and stimulated them with LPS in the presence or absence of IL-4 or IL-13. IL-4 is known to enhance DC production of IL-12 in an IL-4Rα dependent manner, so called “IL-4 DC instruction” [21]–[23]. As expected, BMDCs derived from IL-4Rα^-/lox^ mice and BALB/c wildtype controls had significantly increased IL-12 production after the addition of IL-4 (Figure 1E). In contrast, LPS/IL-4 stimulated BMDCs derived from CD11c^cre^IL-4Rα^-/lox^ mice or from global IL-4Rα^-/-^ mice showed similar levels of IL-12 to those stimulated with LPS alone, with IL-4 having no effect. This demonstrates functional impairment of IL-4Rα signaling on DCs from CD11c^cre^IL-4Rα^-/lox^ mice. In fact, after the addition of LPS alone, BMDCs with a functional IL-4Rα already showed a trend towards increased IL-12p40 levels, suggesting that endogenous levels of IL-4 found in the culture could influence these BMDCs. IL-13 did not increase levels of IL-12, confirming previous DC stimulation studies [22]. As previously reported [29], IL-4 and IL-13 had no significant effect on BMDC maturation, as shown by similar expression of MHCII, CD86, CD80, CD83 and CD40 (data not shown). Total yield of BMDCs per precursor cell seeded was similar in CD11c^cre^IL-4Rα^-/lox^ mice and littermate controls and survival after maturation was not significantly different (data not shown).

### CD11c^cre^IL-4Rα^-/lox^ mice are hypersusceptible to acute *L. major* infection

**Figure 2:**
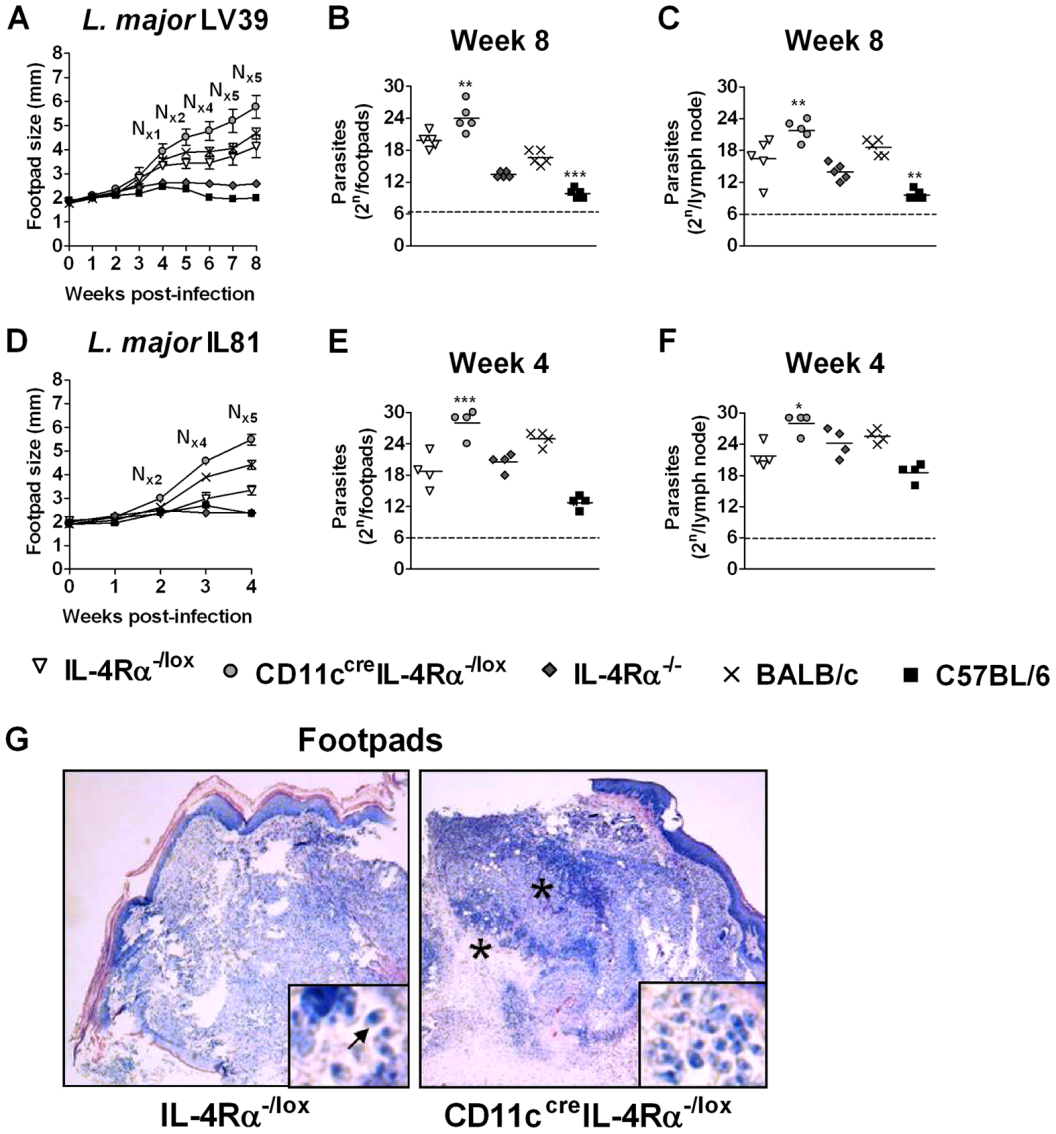
CD11c^cre^IL-4Rα^-/lox^ mice are hypersusceptible to cutaneous *L. major* infection. Mice were infected with *L. major* LV39 (MRHO/SV/59/P) (A to C) or with the more virulent GFP-expressing *L. major* IL81 (MHOM/IL/81/FEBNI) parasite strain (D to F). Footpad swelling was measured at weekly intervals in mice (5 per group) infected subcutaneously with 2×10^6^ stationary phase metacyclic *L. major* promastigotes into the hind footpad (A and D). “N” indicates necrosis or ulceration/mouse. Parasite burden was determined by limiting dilution of single-cell suspensions from homogenized footpads at 8 (B) or 4 week (E) after infection as well as from draining lymph nodes at 8 (C) or 4 week (F) after infection. At week 4 after infection (IL81), formalin-fixed footpads (G) were stained with Giemsa for histopathology (original magnification ×40; asterisks indicate inflammatory foci and insets, arrows indicate amastigote parasites ×800). A representative of two individual experiments is shown with mean values ±SEM. Statistical analysis was performed defining differences to IL-4Rα^-/lox^ mice as significant (*, *p*≤0.05, **, *p*≤0.01; ***, *p*≤0.001).

In order to investigate the role of IL-4Rα signaling on DCs during cutaneous Leishmaniasis, CD11c^cre^IL-4Rα^-/lox^ mice were infected subcutaneously with 2×10^6^ stationary phase metacyclic promastigotes of *L. major* LV39 (MRHO/SV/59/P; Figure 2A, 2B and 2C) or with a more virulent GFP-expressing *L. major* IL81 (MHOM/IL/81/FEBNI; Figure 2D, 2E and 2F) strains into the hind footpad. As previously shown [15], [24], C57BL/6 mice and IL-4Rα^-/-^ deficient BALB/c mice controlled lesion development during acute infection with both *L. major* strains (Figure 2A and 2D), which correlated with low parasite numbers in infected footpads (Figure 2B and 2E) and draining lymph nodes (Figure 2C and 2F). Susceptible WT BALB/c and IL-4Rα^-/lox^ littermate control mice developed progressive footpad swelling after infection with both strains (Figure 2A and 2D), with increased parasite burdens in the infected footpads (Figure 2B and 2E) and draining LN (Figure 2C and 2F). Hemizygous (IL-4Rα^-/lox^ mice) had slightly reduced footpad swelling compared to BALB/c mice in IL81 infection. The greater virulence of IL81 is reflected in more rapid disease progression, with footpad swelling and parasite burden reaching similar levels by 4 weeks to those obtained with LV39 in 8 weeks. Of importance, CD11c^cre^IL-4Rα^-/lox^ mice were hypersusceptible to acute *L. major* infection compared to heterozygous littermate controls and BALB/c mice, showing considerably worsened disease progression when infected with either strain (Figure 2A and 2D), with earlier and dramatically larger footpad lesions, and development of early necrosis (Figure 2A and 2D). Increased disease progression was accompanied by significantly higher parasite numbers in the footpads (Figure 2B and 2E) and LN (Figure 2C and 2F) of infected animals. In addition, infection with a 10-fold lower dose of *L. major* LV39 also resulted in a hypersusceptible phenotype in CD11c^cre^IL-4Rα^-/lox^ mice (Supplementary Figure S1 A–C). Histopathological analysis of CD11c^cre^IL-4Rα^-/lox^ footpads at week 4 after infection with the virulent IL81 revealed severe destruction of epidermis, connective tissue and bone as a result of footpad necrosis, accompanied by increased inflammatory infiltrates and a high load of extracellular *L. major* amastigotes (Figure 2G). In contrast, infected footpads of IL-4Rα^-/lox^ revealed moderate dermal inflammatory infiltrates with mostly intact epidermis, connective tissue and bone. Together, these data reveal that IL-4Rα signaling on DCs play an important role in host protection against acute *L. major* infection.

### A shift towards Th2 responses in CD11c^cre^IL-4Rα^-/lox^ BALB/c mice

**Figure 3:**
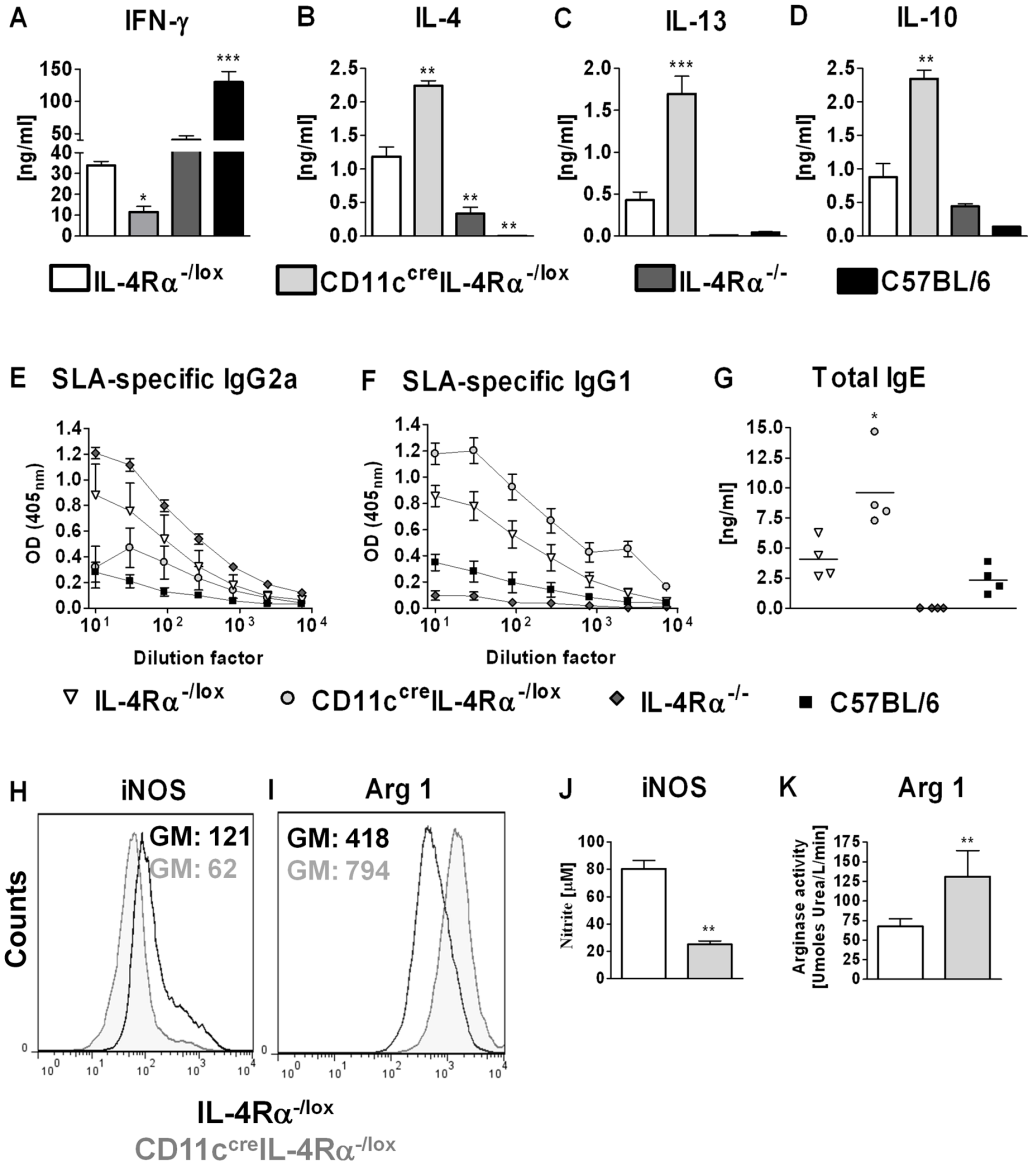
T helper 2 immunity is enhanced in hypersusceptible CD11c^cre^IL-4Rα^-/lox^ mice in response to acute *L. major* IL81 infection. Experimental mice were infected subcutaneously with 2×10^6^ stationary phase metacyclic GFP-expressing *L. major* IL81 promastigotes into the hind footpad (A–D). At week 4 post infection, total CD4^+^ T cells from the draining lymph node were restimulated for 72 hrs with fixed APCs and soluble *Leishmania* antigen (SLA). The production of IFN-γ (A), IL-4 (B), IL-13 (C) and IL-10 (D) was determined by ELISA. (E–G) Antigen-specific IgG2a (E), IgG1 (F) and total IgE (G) antibody production was quantified from infected sera by ELISA. (H–I) Expression of iNOS and arginase 1 in total footpad cells. Total cells were isolated from footpads at week 4 after infection, surface-stained for CD11b^high^MHCII^high^CD11c^−^ macrophages followed by intracellular staining for iNOS (H) and arginase 1 (I). GM = geometric means. (J–K) Production of NO and arginase 1 in total footpad cells. Total cells were isolated from footpads at week 4 after infection and stimulated with 10 ng/ml LPS for 72 h. Production of NO was determined in cell supernatants (J) and cell lysates were assayed for arginase 1 production (K). A representative of two individual experiments is shown with mean values ±SEM. Statistical analysis was performed defining differences to IL-4Rα^-/lox^ mice as significant (*, *p*≤0.05, **, *p*≤0.01; ***, *p*≤0.001).

Th1/Th2-type responses were investigated in CD11c^cre^IL-4Rα^-/lox^ mice and controls during acute cutaneous leishmaniasis (IL81). Antigen-specific restimulation of CD4^+^ T cells sorted from the LN of infected mice and co-cultured with fixed antigen-presenting cells and soluble *Leishmania* antigen (SLA) revealed a significantly reduced IFN-γ response in CD11c^cre^IL-4Rα^-/lox^ mice in comparison to the resistant C57BL/6 or IL-4Rα^-/-^ strains as well as to the susceptible IL-4Rα^-/lox^ littermate controls (Figure 3A). Conversely, the levels of IL-4, IL-13 and IL-10 were significantly higher in CD11c^cre^IL-4Rα^-/lox^ mice compared to IL-4Rα^-/lox^, IL-4Rα^-/-^ and C57BL/6 mice (Figure 3B, 3C and 3D). The observed shift in cytokine responses was confirmed in LN cells, stimulated with anti-CD3 or SLA (data not shown) and systemically in the quality of *Leishmania*-specific antibody immune responses. Sera of week 4 infected mice revealed a predominant type 1 antibody response in IL-4Rα^-/-^ mice, as shown by elevated levels of *Leishmania*-specific IgG2a (Figure 3E). In contrast, CD11c^cre^IL-4Rα^-/lox^ mice displayed a predominant type 2 antibody response shown by marked production of IgG1 and total IgE, which was significantly higher than that observed in littermate IL-4Rα^-/lox^ mice (Figure 3F and 3G). A shift towards Th2-type responses also occurred in CD11c^cre^IL-4Rα^-/lox^ mice in a 10-fold lower dose *L. major* LV39 infection (Supplementary Figure S1 D–H).

As IFN-γ-induced nitric oxide synthase (iNOS) production by classically activated macrophages (caMphs) is a key control mechanism in *L. major* infection [14], the activation state of macrophages was determined in the infected footpad at week 4 after infection. Inflammatory macrophages (CD11b^+^MHCII^+^CD11c^−^) from CD11c^cre^IL-4Rα^-/lox^ mice had significantly reduced iNOS expression compared to those of littermate IL-4Rα^-/lox^ control mice (Figure 3H). Conversely, expression of arginase 1, a marker of alternatively activated macrophages (aaMphs), was higher in macrophages of CD11c^cre^IL-4Rα^-/lox^ mice (Figure 3I). This altered phenotype was confirmed in iNOS and arginase activity assays performed on total footpad cells stimulated with LPS (Figure 3J and 3K). Together, these results demonstrate a shift towards Type 2 responses and reduced macrophage effector functions in CD11c^cre^IL-4Rα^-/lox^ mice.

### CD11c^cre^IL-4Rα^-/lox^ mice have increased parasite loads in peripheral organs

**Figure 4:**
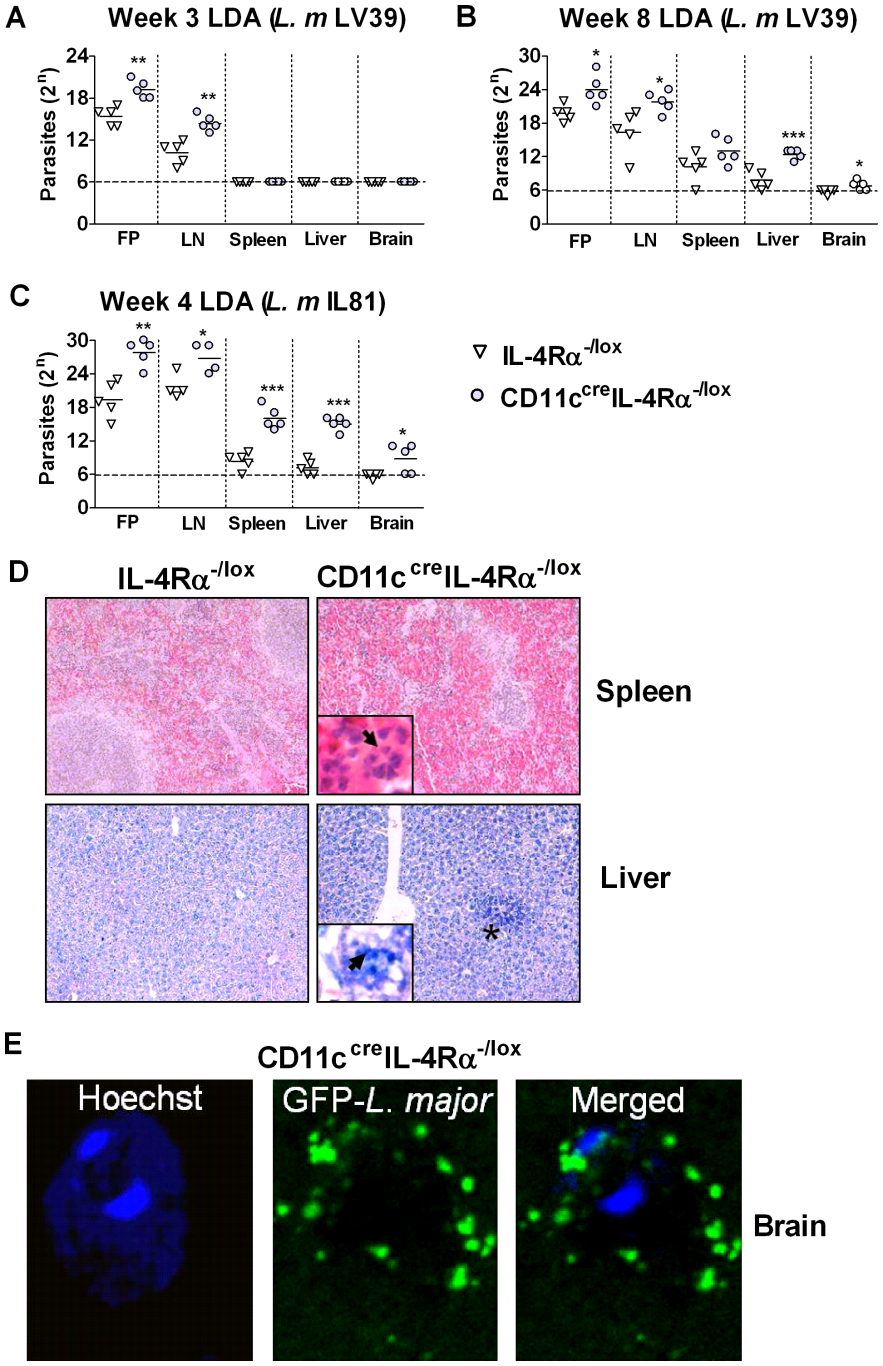
Impairment of IL-4Rα signaling *in vivo* results in increased *L. major* parasite loads in peripheral organs. CD11c^cre^IL-4Rα^-/lox^ and littermate mice were infected subcutaneously with 2×10^6^ stationary phase metacyclic *L. major* (*L. m* LV39) promastigotes into the hind footpad. Parasite load was determined by limiting dilution assay (LDA) of single-cell suspensions from homogenized footpad, lymph node, spleen, liver and brain at week 3 (A) and week 8 (B) after infection. Similarly, organs were harvested from mice infected with GFP-expressing *L. major* (*L. m* IL81) at week 4 after infection for limiting dilution assay (C). At the same time point, histopathology was analysed using formalin-fixed spleen and liver (D) stained with H&E and Giemsa, respectively (original magnification ×100; asterisks indicate inflammatory foci and insets, arrows indicate amastigote parasites ×800). Frozen brain sections (E) were stained with Hoechst nuclear stain (blue) and visualized by confocal microscopy for the presence of GFP-*L. major* amastigote parasites (original magnification ×400). A representative of two individual experiments is shown with mean values ±SEM. Statistical analysis was performed defining differences to IL-4Rα^-/lox^ mice (*, *p*≤0.05, **, *p*≤0.01, ***, *p*≤0.001).

In *L. major* LV39 infection, parasites were present only in footpads and the draining lymph nodes at week 3, whereas by week 8 parasites had disseminated to the spleen and liver in both CD11c^cre^IL-4Rα^-/lox^ mice and littermate controls (Figure 4A and 4B). Parasite burdens were much higher in the organs of infected CD11c^cre^IL-4Rα^-/lox^ mice, compared to littermate control mice. Moreover, in some CD11c^cre^IL-4Rα^-/lox^ mice, but not in control mice, *L. major* parasites had disseminated as far as the brain by week 8 after infection (Figure 4B). Similar disease progression was observed after infection with *L. major* IL81 (Figure 4C), where CD11c^cre^IL-4Rα^-/lox^ mice already displayed noticeable splenomegaly at 4 weeks post infection (data not shown), and had strikingly increased parasite burdens in all organs analyzed, including the brain (Figure 4C). Histological analysis confirmed the increased presence of disseminated parasites in the spleen and liver of CD11c^cre^IL-4Rα^-/lox^ mice (IL81, week 4), as shown by the high load of extracellular *L. major* amastigotes (spleen) and the prevalence of inflammatory foci and leishmanial bodies in mononuclear cells (liver) (Figure 4D). The presence of parasites in brains of perfused CD11c^cre^IL-4Rα^-/lox^ mice (IL81, week 4) was also confirmed by confocal microscopy (Figure 4E). Parasites were not visible in the brains of littermate controls (data not shown). These results demonstrate a drastic increase in numbers of disseminated parasites in peripheral organs of infected CD11c^cre^IL-4Rα^-/lox^ mice. Although it has been reported that dissemination could occur within hours after high-dose parasite inoculation [30], infection with GFP^+^ *L. major* IL81 and analysis by flow cytometry demonstrated that GFP^+^ parasites was not detectable in the spleen at 1 or 3 days post infection, whereas at week 4 there was an increase in GFP^+^ cells compared to day 0 (Supplementary Figure S2).

### IL-4Rα-deficient DCs are infected in LN and spleen at week 4 after *L. major* IL81 infection and have impaired killing effector functions

**Figure 5:**
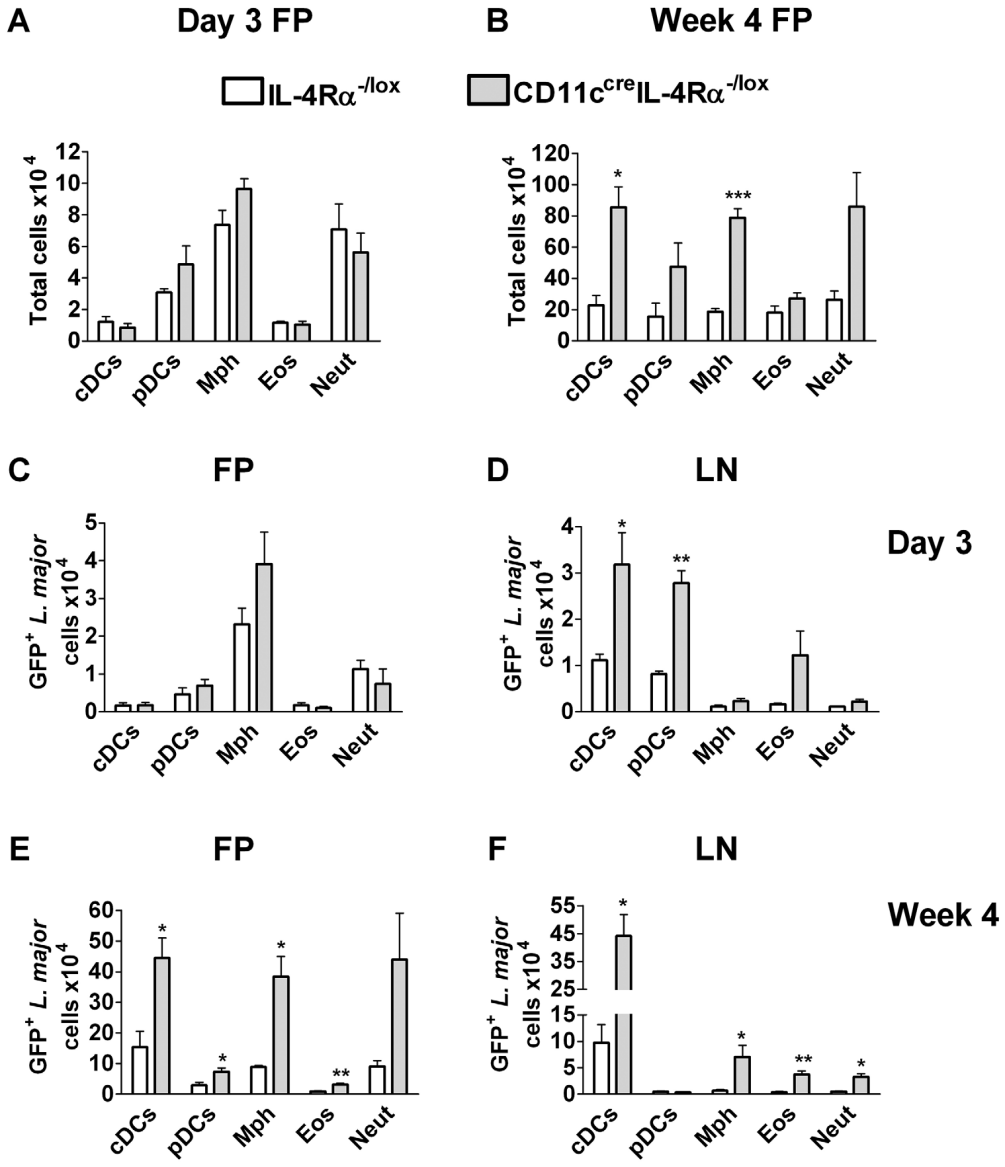
Cell populations infected with *L. major* parasites. CD11c^cre^IL-4Rα^-/lox^ and littermate mice were infected subcutaneously with 2×10^6^ stationary phase metacyclic GFP-expressing *L. major* IL81 promastigotes into the hind footpad. (A–B) Total cells from footpads were analysed for different cell populations at day 3 (A) and week 4 (B) after infection. (C–F) Number of GFP^+^ *L. major* parasites was identified within indicated cell populations derived from footpad (C, E) and lymph node (D, F) at day 3 and week 4 after infection, respectively. Cell populations were differentiated based on the following markers; conventional dendritic cells (cDCs; CD11c^high^MHCII^high^), plasmacytoid DCs (pDCs; CD11c^+^PDCA^+^SiglecH^+^), macrophages (Mph; CD11b^high^MHCII^high^CD11c^−^), Eosinophils (Eos; SiglecF^+^CD11c^−^), Neutrophils (Neut; GR-1^high^SSC^high^FSC^high^ CD11c^−^). Data is expressed as mean ± SEM. Statistical analysis was performed defining differences to IL-4Rα^-/lox^ mice (*, *p*≤0.05, **, *p*≤0.01, ***, *p*≤0.001). FP = Footpad and LN = Lymph node.

In order to determine if dendritic cells could harbor *L. major* parasites, GFP-expressing *L. major* parasites (IL81) were used to track infected cell populations in different organs by flow cytometry at different time-points (day 3, day 7 and week 4) after infection. Parasite replication occurred in GFP^+^ cell populations that were sorted and plated out for limiting dilution assays, indicating that GFP positivity was a good marker for viable parasites associated with cells (Supplementary Figure S3). At day 3 after GFP-*L. major* IL81 infection, plasmacytoid DCs (pDCs), macrophages and neutrophils had infiltrated the infected footpad (Figure 5A). By 4 weeks post infection, numbers of infiltrating cells had increased substantially, with conventional DCs (cDCs) also now present in high numbers (Figure 5B). The number of infiltrating cells was significantly higher in CD11c^cre^IL-4Rα^-/lox^ mice compared to IL-4Rα^-/lox^ mice (Figure 5B). At the early time point in FP, macrophages were infected with GFP^+^ Leishmania, with similar numbers in CD11c^cre^IL-4Rα^-/lox^ mice and littermate controls (Figure 5C). This was in contrast to the draining lymph node, where conventional and plasmacytoid DCs were infected, with higher numbers of DCs infected in CD11c^cre^IL-4Rα^-/lox^ mice compared to controls (Figure 5D). Similar results were obtained at day 7 post-infection (data not shown). At week 4 post infection, the footpad harbored a pool of infected cells, namely macrophages, cDCs and neutrophils (Figure 5E), while in the draining lymph node, the cDCs were still infected compared to the other cell types (Figure 5F). Again the number of infected DCs was significantly higher in CD11c^cre^IL-4Rα^-/lox^ mice (Figure 5E and 5F) compared to littermate controls. However, overall numbers of DCs infiltrating the LN at week 4 after *L. major* IL81 infection were similar in both CD11c^cre^IL-4Rα^-/lox^ mice and littermate control mice (data not shown), suggesting that differences in parasite killing and not DC migration were responsible for the increased number of infected DCs in CD11c^cre^IL-4Rα^-/lox^ mice.

**Figure 6:**
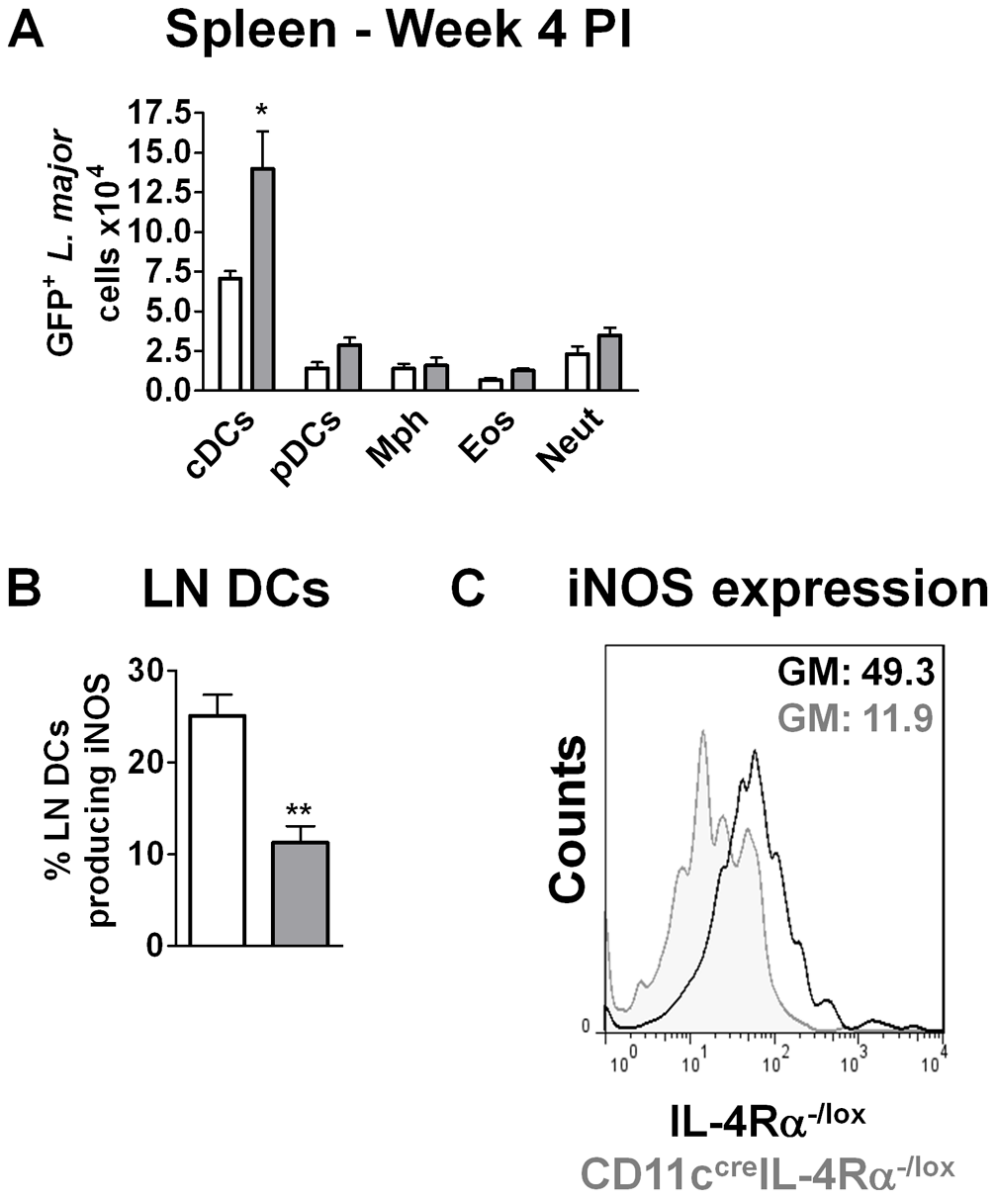
Infected DCs in CD11c^cre^IL-4Rα^-/lox^ mice have reduced iNOS production. CD11c^cre^IL-4Rα^-/lox^ and littermate mice were infected subcutaneously with 2×10^6^ stationary phase metacyclic GFP-expressing *L. major* IL81 promastigotes into the hind footpad. (A) At week 4 after infection, spleens were harvested to analyse number of GFP^+^ *L. major* parasites within the indicated cell populations. Cell populations were differentiated based on the following markers; conventional dendritic cells (cDCs; CD11c^high^MHCII^high^), plasmacytoid DCs (pDCs; CD11c^+^PDCA^+^SiglecH^+^), macrophages (Mph; CD11b^high^MHCII^high^CD11c^−^), Eosinophils (Eos; SiglecF^+^CD11c^−^), Neutrophils (Neut; GR-1^high^SSC^high^FSC^high^ CD11c^−^). (B) Percentage of DCs producing iNOS. Total lymph node cells were surface-stained for CD11c^high^MHCII^high^ DCs followed by intracellular staining for percent iNOS-producing DCs. (C) Histogram plots showing intracellular iNOS expression in CD11c^high^MHCII^high^ DCs. Data is expressed as mean ± SEM. Statistical analysis was performed defining differences to IL-4Rα^-/lox^ mice (*, *p*≤0.05, **, *p*≤0.01).

Infected DCs were also found in the spleen, with significantly increased numbers of infected cells in CD11c^cre^IL-4Rα^-/lox^ mice compared to controls (Figure 6A). However, overall numbers of DCs infiltrating the spleen were also increased to a similar degree in both CD11c^cre^IL-4Rα^-/lox^ mice and littermate controls at week 4 (data not shown), again suggesting that differences in parasite killing and not DC migration were responsible for the increased parasite loads in CD11c^cre^IL-4Rα^-/lox^ mice. Although it is well known that iNOS-mediated NO production in classically-activated macrophages drives intracellular killing of *L. major* parasites, a recent study has now implicated a population of iNOS^+^ – producing inflammatory DCs in controlling *Leishmania* infection [31]. We therefore examined iNOS production by DCs in CD11c^cre^IL-4Rα^-/lox^ and littermate control mice using intracellular FACS. In hypersusceptible CD11c^cre^IL-4Rα^-/lox^ mice, a significantly reduced percentage of CD11c^high^MHCII^high^ DCs produced iNOS compared to DCs from IL-4Rα^-/lox^ littermate control mice (Figure 6B). This was confirmed at the level of intracellular NO expression, which was also reduced in DCs from CD11c^cre^IL-4Rα^-/lox^ mice (Figure 6C). Together, these data demonstrate that DCs from CD11c^cre^IL-4Rα^-/lox^ mice have reduced NO killing effector functions, further explaining the increased parasite burdens in the DCs of these mice.

### IL-4Rα-deficient DCs have impaired DC instruction during infection *in vivo*

**Figure 7:**
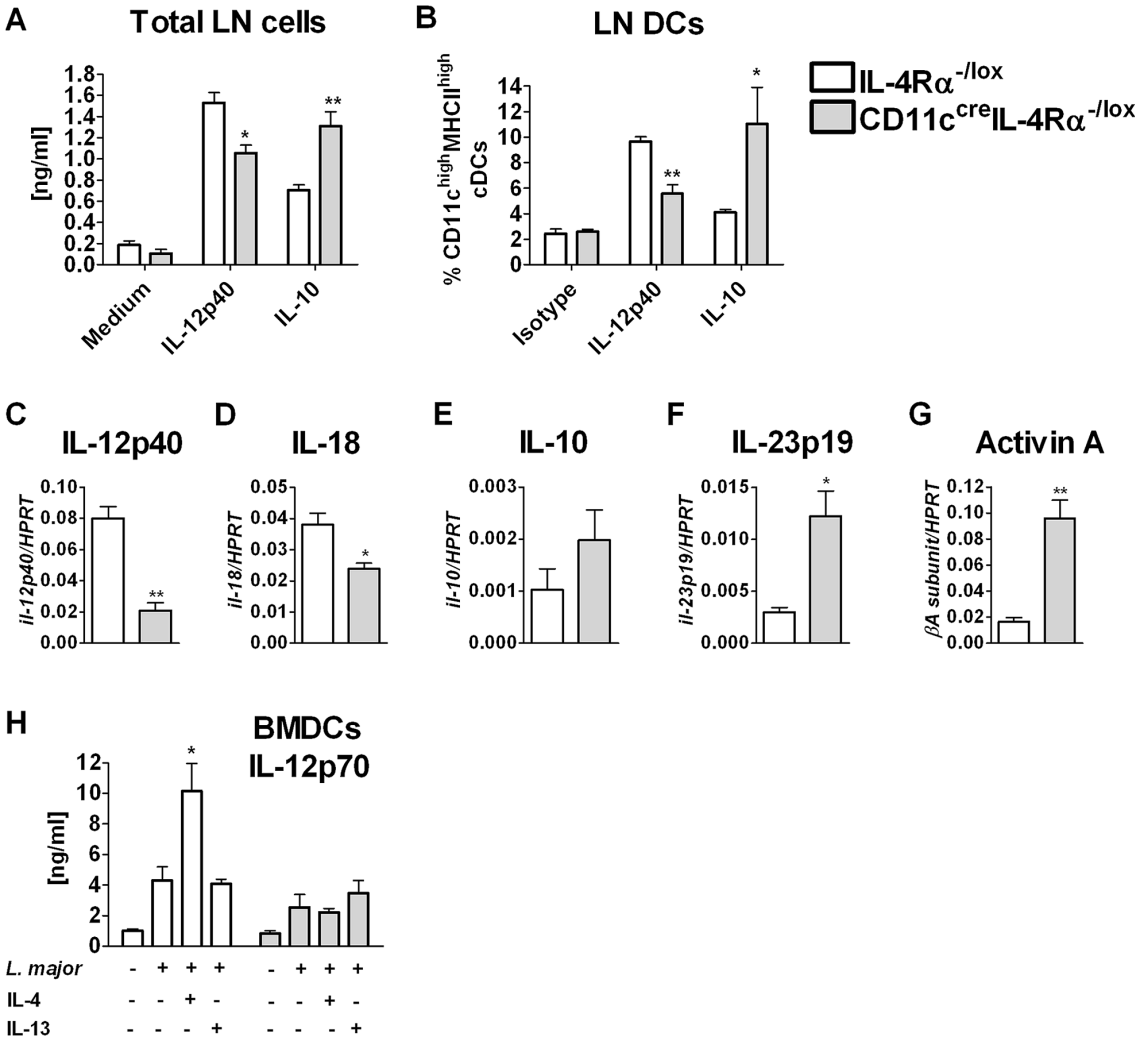
Abrogation of IL-4Rα expression in dendritic cells impairs dendritic cell instruction and alters DC phenotype *in vivo*. Mice were infected subcutaneously with 2×10^6^ stationary phase metacyclic GFP-*L. major* IL81 promastigotes into the hind footpad. (A) After 4 weeks of infection, total lymph node cells were restimulated with SLA and production of IL-12p40 and IL-10 was determined by ELISA. (B) Intracellular staining of IL-12p40 and IL-10 in lymph node dendritic cells following incubation with PMA/Ionomycin/Monensin for 4 h at 37°C. Percent cytokine producing cells are shown. mRNA expression of IL-12p40 (C), IL-18 (D), IL-10 (E), IL-23p19 (F) and Activin A (G) was determined by real-time RT-PCR from sorted LN dendritic cells. Expression was normalised against the housekeeping gene *HPRT*. (H) IL-12p70 production from BMDCs infected with *L. major* in the presence or absence of rIL-4 or rIL-13. Culture supernatants were collected after 48 hours to determine IL-12p70 levels by ELISA. Data is expressed as mean ±SEM. Statistical analysis was performed defining differences to IL-4Rα^-/lox^ mice (*, *p*≤0.05, **, *p*≤0.01, ***, *p*≤0.001).

Previous studies using BMDCs found that IL-4-mediated instruction results in reduced IL-10 production that is responsible for increased IL-12p40 production by DCs upon stimulation with IL-4 plus CpG or LPS [21], [23]. To test whether endogenous amounts of IL-4 could mediate DC instruction *in vivo*, CD11c^cre^IL-4Rα^-/lox^ mice and controls were infected with *L. major* IL81. At 4 weeks post infection, total LN cells were restimulated with SLA and cytokines were measured in the supernatant. Lymph node cells from infected CD11c^cre^IL-4Rα^-/lox^ mice produced significantly reduced IL-12p40 but increased IL-10 compared to littermate IL-4Rα^-/lox^ mice (Figure 7A). Moreover, intracellular cytokine staining revealed that DCs from CD11c^cre^IL-4Rα^-/lox^ mice produced less IL-12p40 and more IL-10 than those from littermate IL-4Rα^-/lox^ controls (Figure 7B and Figure S4). Quantification of mRNA found decreased expression of the Th1-promoting cytokine genes for IL-12p40 (Figure 7C) and IL-18 (Figure 7D) in sorted LN DCs from CD11c^cre^IL-4Rα^-/lox^ mice compared to controls. In contrast, there was a trend towards increased mRNA expression of IL-10 as well as significantly increased mRNA expression of IL-23 and activin A, cytokines which are involved in inducing Th2 responses by promoting Th17 and alternative activation of macrophages, respectively [32], [33] (Figure 7E–G). In addition, differences in IL-12p70 production were detected *in vitro*. *L. major*/IL-4 stimulated BMDCs derived from IL-4Rα^-/lox^ mice showed increased IL-12p70 production, whereas IL-4 had no additive effect on IL-12p70 production in BMDCs from CD11c^cre^IL-4Rα^-/lox^ mice (Figure 7H). IL-13 did not increase IL-12p70 production, as previously shown [22].

## Discussion

Understanding mechanisms of immune control in cutaneous Leishmaniasis is critical for the design of effective therapeutics and vaccines. Although several studies have clearly established that IL-4 is a key cytokine in the development of non-healing disease in BALB/c mice [8], [19], [34], [35], apparently contradictory evidence also suggests that IL-4 has the ability to instruct protective Th1 responses [21], [36]–[41]. The term “instruction theory” was coined when IL-4 was found to promote increased production of IL-12 by BMDCs [20]–[22]. IL-4, but not IL-13, enhances the production of IL-12 induced by pathogen products via signalling through the type 1 IL-4 receptor [21], [22]. The mechanism behind instruction was found to be inhibition of IL-10 by IL-4, leading to higher levels of IL-12 and increased protective Th1 responses [23]. Several studies also indicate that IL-4 and IL-13 may play a role in promoting DC maturation [22], [42]. However, most *in vitro* and *in vivo* studies on the effects of IL-4 and IL-13 on DCs have been conducted with exogenously administered IL-4 or IL-13, and thus the relevance of biological quantities of IL-4 signalling through IL-4Rα on DCs during disease *in vivo* has not been demonstrated. To address these issues, dendritic cell-specific (CD11c^cre^IL-4Rα^-/lox^) BALB/c mice were generated using the cre/*lox*P recombinase system under control of the *cd11c* locus. These mice were found to have abrogated IL-4Rα expression on DCs and alveolar macrophages, with other cell types maintaining IL-4Rα expression and functioning.

Infection of CD11c^cre^IL-4Rα^-/lox^ mice with *L. major* LV39 and IL81 revealed IL-4Rα signaling on DCs to be highly important in protection against cutaneous Leishmaniasis. Compared to IL-4Rα^-/lox^ littermate controls, CD11c^cre^IL-4Rα^-/lox^ mice showed dramatically worsened disease progression, with increased footpad swelling and necrosis, and significantly higher parasite burdens both locally and in visceral organs such as the spleen and liver. As expected, genetically resistant C57BL/6 mice effectively controlled infection, as did global IL-4Rα^-/-^ mice, which have been shown to be resistant during the acute phase of *L. major* infection, with disease progression in the chronic phase only [15], [24]. Progressive disease during *L. major* infection in BALB/c mice has been attributed to the predominance of Th2 cytokines and type 2 antibody immune responses [8], [9], [11], with a previous study by our laboratory showing that CD4^+^ T cell specific IL-4Rα deficient mice were highly resistant to *L. major* infection [24]. Analysis of CD4^+^ T cell cytokine responses in CD11c^cre^IL-4Rα^-/lox^ mice revealed a decrease in IFN-γ accompanied by a marked increase in IL-4, IL-13 and IL-10, while increased secretion of IgG1 and IgE by B cells confirmed a shift towards a Th2-type immune phenotype. Aside from its role in instruction, IL-10 is known to be a susceptibility factor for *L. major* infection, being produced at higher levels in susceptible BALB/c mice and capable of suppressing Th1-mediated effector functions [3], [43]. In humans, IL-10 is strongly associated with persistent infection [44].

IFN-γ plays an important role in mediating protective immunity during *L. major* infection by classically-activating macrophages to induce nitric oxide synthase-mediated NO production for intracellular killing of parasites [9], [14], [45], [46]. Latent Leishmaniasis is reactivated in chronically infected healthy C57BL/6 mice by inhibition of endogenous NOS-2, indicating that iNOS expression is crucial for the sustained control of *L. major* infection [9], [31], [47]. Induction of iNOS-mediated NO production is counter-regulated by IL-4/IL-13 and IL-4Rα, which promote the development of alternatively activated macrophages and arginase 1 production through depletion of L-arginine as a substrate for iNOS. Interestingly, IL-10 has also been shown to suppress intracellular killing of pathogens in macrophages by suppressing IFN-γ responses [48]–[50] and can induce an alternatively activated macrophage type phenotype in the absence of IL-4 and IL-13 [51]. Parasites such as *Leishmania* can utilize polyamines generated by arginase 1 activity for their own growth, making alternatively activated macrophages a favorable environment for their survival [52]–[54]. Accumulating reports have demonstrated a role for alternative macrophage activation and arginase 1 expression in influencing susceptibility to *L. major* infection [7], [9], [55], [56]. LysM^cre^IL-4Rα^-/lox^ mice which lack IL-4/IL-13 induced alternative activation of macrophages were found to have increased resistance to infection [9], while neutralization of endogenous arginase 1 with N-hydroxy-nor-L-arginine leads to complete healing in BALB/c mice [55].

Macrophages from the footpads of CD11c^cre^IL-4Rα^-/lox^ mice were found to have reduced iNOS expression and increased arginase 1 expression compared to those from littermate control IL-4Rα^-/lox^ mice, demonstrating a shift in macrophage effector function most likely as a consequence of increased IL-4, IL-13 and IL-10. Recently it has been shown that DCs can also become alternatively activated by upregulating markers such as Ym-1 and RELM-α after administration of IL-4 [29]. In our study, the data suggest that IL-4Rα-independent alternative activation of DCs is also possible, as DCs from CD11c^cre^IL-4Rα^-/lox^ had decreased iNOS expression, possibly a consequence of reduced IFN-γ and/or increased IL-10 and activin A, and had higher parasite loads than those from littermate controls. Previous studies have revealed that iNOS-producing DCs constitute a major Th1-regulated effector cell population and contribute to resistance to infection by *L. major* [31], *L. monocytogenes* [57] and *Brucella* spp. [58]. The reduced ability of both macrophages and DCs to initiate NO-mediated killing of *L. major* in CD11c^cre^IL-4Rα^-/lox^ mice is therefore likely to play a role in the uncontrolled parasite replication observed both in the footpad and at peripheral sites.

In susceptible BALB/c mice, *L. major* parasites can disseminate within 24 hours from the site of infection in the footpad to the popliteal lymph nodes, spleen, liver, lungs and bone marrow [30], [59]. However, *L. major* parasites were not detected at early time points during IL81 infection (day 1 and day 3) but were detected at week 4, and were also detected at week 8 but not at week 3 during LV39 infection, suggesting that parasite dissemination may have occurred at a later stage of infection. Dissemination is inhibited by the administration of recombinant IL-12 and resistant mouse strains restrict the spread of the parasites [30]. While several susceptible mouse strains have been reported to show some increase in dissemination [60]–[62], disseminated parasite loads in CD11c^cre^IL-4Rα^-/lox^ mice were unusually dramatic, with relatively higher parasite burdens in the spleens and footpads compared to other susceptible strains. Unexpectedly, parasites were even identified within the brain of some of the CD11c^cre^IL-4Rα^-/lox^ mice. This suggests that the *L. major* parasites managed to cross the immunological blood-brain barrier, which has only rarely been reported for this cutaneous strain with very low levels of parasites detected [63]. However, dissemination of parasites to the central nervous system (CNS) has been frequently observed in visceral Leishmaniasis in both humans and dogs [64]–[67]. It has been suggested that parasites arrive in the CNS via infected leukocytes [65] and/or disruption to the blood brain barrier caused by inflammation [67]. Studying the mechanisms by which other pathogens, such as bacteria, invade the CNS may lend insights into *Leishmania* dissemination. Many intracellular organisms such as *Mycobacterium tuberculosis*, *Listeria monocytogenes*, *Brucella* spp. and *Salmonella* spp. appear to make use of the “Trojan-horse” mechanism, using phagocyte facilitated invasion for entry into the CNS [68]. After infection with intracellular pathogens, phagocytes undergo phenotypical changes, such as increased migratory activity and increased expression of adhesion molecules and proinflammatory cytokines, all of which could aid in dissemination and crossing of the blood-brain barrier [68], [69]. Whether infected phagocytes are recruited to the CNS by specific or non-specific means is unknown [69]. In order to determine which cells were infected by *L. major*, mice were infected with GFP-IL81 parasites and cell populations containing parasites were identified by flow cytometry.

At day 3 and 7 after infection, macrophages harbored *L. major* in the footpad, while pDCs and cDCs were found to be infected in the lymph node. Similar to other reports, this indicates that DCs were responsible for transporting parasites to the lymph node [70]. At week 4, *L. major* parasites were still detected in macrophages in the footpads, as well as in DCs and neutrophils, but in the LN they were primarily found in DCs. The number of infected DCs in both footpad and LN was significantly higher in CD11c^cre^IL-4Rα^-/lox^ mice. A previous study also reported that DCs were the primary infected cell population in the draining LN of *L. major* infected mice [70]. DCs were also infected with *L. major* parasites in the spleen, with CD11c^cre^IL-4Rα^-/lox^ mice again showing a greater number of infected DCs. Numbers of DCs infiltrating the LN and spleen were equivalent in both CD11c^cre^IL-4Rα^-/lox^ mice and littermate controls during infection. This suggests that the increased survival and/or growth of parasites in DCs, as a consequence of significantly reduced DC iNOS production, was responsible for the increase in infected cell numbers in CD11c^cre^IL-4Rα^-/lox^ mice. Interestingly, a recent study found that infected DCs, which are monocyte-derived CD11b^+^ inflammatory DCs expressing Ly6C, F480, Ly6G and iNOS, showed a unique ability to disseminate to peripheral sites in *M. tuberculosis* infection [71]. Furthermore, CD11b^+^Ly6C^+^ cells were found to be the principal phagocytic cells harboring *L. monocytogenes* in circulation [69], [72]. We hypothesize that dendritic cells may therefore play a role in disseminating *L. major* parasites to peripheral sites and that their killing effector responses could be important in controlling disease.

The reduced Th1 and increased Th2 responses in CD11c^cre^IL-4Rα^-/lox^ mice suggests that instruction theory is relevant *in vivo*, and more importantly, that biological quantities of IL-4 acting through DCs can promote resistance to *Leishmania* infection. DCs from lymph nodes of CD11c^cre^IL-4Rα^-/lox^ mice produced more IL-10 and less IL-12 than those from IL-4Rα^-/lox^ mice. Quantification of mRNA expression also revealed interesting differences in DCs from CD11c^cre^IL-4Rα^-/lox^ mice. Expression of the Th1-promoting genes for IL-12p40 and IL-18 was decreased compared to DCs from littermate control mice, while expression of the Th2-promoting genes for IL-23p19 and activin A were significantly increased. IL-23 production by DCs has been shown to promote Th17 [32], leading to increased neutrophils that enhance susceptibility to *L. major* by acting as Trojan horses [73]. Activin A is a pleiotropic cytokine belonging to the TGF-beta superfamily, and has previously been found to promote alternative activation of macrophages by inducing Arginase 1 and decreasing IFN-γ-induced expression of iNOS [33]. The absence of IL-4Rα signalling on DCs therefore appears to have a more complex influence on the dendritic cells than just affecting IL-12 production during cutaneous Leishmaniasis *in vivo*.

Dendritic cell instruction may not be restricted to Leishmaniasis, since other disease models have also demonstrated a protective role for IL-4. Experimental infections with *Candida albicans* in IL-4 deficient mice led to impaired development of Th1 responses [38], and a Th1 promoting effect of IL-4 has also been observed in autoimmunity [36], [40], [74], tumor immunity [39], [75], [76] and contact sensitivity reactions [41], [77]. There is also evidence to suggest that IL-4 may promote Th1 development in humans, since both human and mouse DCs produce increased levels of bioactive IL-12 after stimulation with IL-4 [20]. A similar effect was observed in human peripheral blood mononuclear cells (PBMCs) treated with IL-4 plus lipopolysaccharide or *Staphylococcus aureus* [78]. Incorporating exogenous IL-4 as an adjuvant for enhancing strong Th1 responses could therefore be utilised to boost vaccine efficiency against cutaneous Leishmaniasis. Accordingly, parallel studies examining the efficacy of IL-4 as an adjuvant during BMDC-mediated vaccination against *L. major*, found that IL-4 instruction of DCs was critical in eliciting protective immune responses [79]. The role of IL-4Rα signalling on DCs in eliciting immunity to other intracellular pathogens is therefore of interest to vaccination strategies, and an exciting avenue to be explored.

## Materials and Methods

### Generation and genotyping of CD11c^cre^IL-4Rα^-/lox^ BALB/c mice

CD11c^cre^ mice [27] were crossed with IL-4Rα^lox/lox^ BALB/c mice [28] and complete IL-4Rα^-/-^ BALB/c mice [15] to generate hemizygous CD11c^cre^IL-4Rα^-/lox^ mice. Mice were backcrossed to a BALB/c background for 9 generations to generate CD11c^cre^IL-4Rα^-/lox^ BALB/c mice. Hemizygous littermate controls (IL-4Rα^-/lox^) were used as controls in all experiments. Mice were genotyped as described previously [28]. All mice were housed in specific-pathogen free barrier conditions in individually ventilated cages. Experimental mice were age and sex matched and used between 8–12 weeks of age.

### Ethics statement

This study was performed in strict accordance with the recommendations of the South African national guidelines and University of Cape Town of practice for laboratory animal procedures. All mouse experiments were performed according to protocols approved by the Animal Research Ethics Committee of the Health Sciences Faculty, University of Cape Town (Permit Number: 009/042). All efforts were made to minimize suffering of the animals.

### Analysis of IL-4Rα deletion efficiency

Genomic DNA was isolated from spleen DCs (CD11c^+^MHCII^+^) sorted using a FACS Vantage flow cytometer (BD Immunocytometry systems). Purity was determined by flow cytometry and checked by cytospin and staining with the Rapidiff Stain set (Clinical Diagnostics CC, Southdale, South Africa) and was at least 99%. A standard curve was prepared from serial 10-fold DNA dilutions of cloned IL-4Rα exon 5 and exon 8 DNA and RT-PCR was performed using the following primers; exon 5: forward 5′ AACCTGGGAAGTTGTG 3′ and reverse 5′ CACAGTTCCATCTGGTAT 3′, exon 8: forward 5′ GTACAGCGCACATTGTTTTT 3′ and reverse 5′ CTCGGCGCA CTGACCCATCT 3′.

### Flow cytometry

The following antibodies were used for flow cytometry: SiglecF-PE, CD11c-APC, MHCII-APC, F480-PE, CD11b-FITC, CD3-FITC, CD19-PE, PDCA-APC, SiglecH-PE, CD11b-PE, CD11c-PE, CD4-PerCP, CD8-PE, GR-1-PE, CD3-PerCP, anti-CD124-PE, rat anti-mouse IgG2a-PE, CD11c-biotin, CD103-biotin, CD124-biotin and rat-anti-mouse IgG2a biotin with streptavidin-APC (all BD Bioscience, Erembodegem, Belgium) and MHCII-biotin with PerCP streptavidin (BD Bioscience). For intracellular cytokine staining, popliteal lymph node cells from *L. major* infected mice were seeded at 2×10^6^ cells/well and stimulated at 37°C for 4 hours with phorbal myristate acetate (Sigma-Aldrich) (50 ng/ml), ionomycin (Sigma-Aldrich) (250 ng/ml) and monensin (Sigma-Aldrich) (200 µM) in DMEM/10% FCS. Dendritic cells were stained with CD11c-PE-Cy7 (BD Bioscience) and MHCII-APC, fixed and permeabilized, and intracellular cytokines were stained with anti-IL-10, anti-IL-12 and isotype controls (BD Bioscience) (all PE-labelled). Cells were acquired on a FACS Calibur machine (BD Immunocytometry systems, San Jose, CA, USA) and data were analyzed using Flowjo software (Treestar, Ashland, OR, USA).

### IL-4Rα responsiveness in bone marrow-derived dendritic cells (BMDCs)

BMDCs were generated from bone-marrow progenitors of CD11c^cre^IL-4Rα^-/lox^ and littermate control mice using 200 U/ml recombinant mouse granulocyte-macrophage colony-stimulating factor (GM-CSF) (Sigma-Aldrich) as previously described [80]. On Day 10, non-adherent cells were harvested and 5×10^5^ BMDCs were stimulated with LPS (Sigma-Aldrich; 1 µg/ml) or *Leishmania major* IL-81 promastigotes (MOI: 10 parasites/cell) in the presence or absence of 1000 U/ml recombinant mouse IL-4 or IL-13 (rIL-4/rIL-13, BD Biosciences) for 48 h. Following incubation, levels of IL-12p40, IL-12p70 and IL-10 were measured in culture supernatants by ELISA as previously described [15].

### ELISAs

Cytokines in cell supernatants were measured by sandwich ELISA as previously described [15]. For antibody ELISAs, blood was collected in serum separator tubes (BD Bioscience, San Diego, CA). Antigen-specific IgG1, IgG2a and IgG2b were quantified by ELISA, as previously described [15]. Detection limits were 5 ng/ml for IgG1 and IgG2b and 0.1 ng/ml for IgG2a and IgG3. Total IgE was determined as described [15]. The detection limits was 8 ng/ml for total IgE.

### *Leishmania major* infection

*L. major* LV39 (MRHO/SV/59/P) and GFP-expressing *L. major* IL81 (MHOM/IL/81/FEBNI) (kind gift from Prof. Heidrun Moll, University of Würzburg, Germany) strains were maintained by continuous passage in BALB/c mice and prepared for infection as described previously [15]. Anaesthetised mice were inoculated subcutaneously with 2×10^6^ or 2×10^5^ stationary phase metacyclic promastigotes into the left hind footpad in a volume of 50 µl of HBSS (Invitrogen). Swelling of infected footpads was monitored weekly using a Mitutoyo micrometer calliper (Brütsch, Zürich, Switzerland).

### Histology

Footpads, spleens and livers were fixed in 4% formaldehyde in phosphate buffered saline and embedded in wax. Tissue sections were stained with either haemotoxylin and eosin or Giemsa.

### Immunohistochemistry

Following infection of mice with GFP-*L. major* IL81 parasites for 4 weeks, isolated brain tissue was immediately embedded in OCT (Tissue-Tek; Sakura, Zoeterwoude, Netherlands) medium. Pre-fixing of tissues was avoided to minimize background staining from the fixative. OCT-embedded brain tissue were cut into 10 µm frozen sections and mounted on 3-aminopropyltriethoxysilane-coated slides. Following acetone fixation of tissue, sections were stained with nuclear stain Hoechst. Coverslips were then mounted on sections using Mowiol 4–88 mounting medium (Calbiochem) with anti-fade (Sigma). Images were acquired and analyzed by Ziess LSM 510 confocal microscope (Jena, Germany).

### Detection of viable parasite burden

Infected organ and tissue cell suspensions were cultured in Schneider's culture medium (Sigma). Prior to removal of mouse brain tissue for detection of parasite burden, animals were perfused with 20 ml sterile saline solution. Detection of viable parasite burden was estimated by two-fold limiting dilution assay as previously described [15].

### Antigen-specific restimulation

CD4^+^ T cells were positively selected using anti-CD4 MACS beads (Miltenyi Biotec) according to the manufacturer's instructions (purity >95%). Thy1.2-labeled splenocytes were T cell depleted by complement-mediated lysis to enrich antigen presenting cells (APCs). APCs were fixed with mitomycin C (50 µg/ml, 20 min at 37°C) and washed extensively in complete IMDM. A total of 2×10^5^ purified CD4^+^ T cells and 1×10^5^ APCs were cultured with SLA (50 µg/ml). After 72 h incubation at 37°C, supernatants were collected and cytokine production analysed as previously described [28].

### Isolation of footpad and spleen cells

Muscle tissue was separated from infected footpads and digested in DMEM medium supplemented with Collagenase IV (Sigman-Aldrich; 1 mg/ml) and DNase I (Sigma-Aldrich; 1 mg/ml) at 37°C for 60 min. Following incubation, single cell suspensions were isolated by straining through 40 µM cell-strainers. Spleen cells were isolated by pressing through 70 µM cell-strainers, red blood cell lysis was performed and white blood cells were washed and resuspended in 10% DMEM (Gibco).

### Cell sorting

Total lymph node or footpad cells were labeled with specific mAbs and populations isolated by cell sorting on a FACS Vantage machine. Macrophages from the footpad were gated as CD11b^high^MHCII^high^CD11c^−^ cells and DCs, macrophages, neutrophils and B cells from the lymph node were gated as CD11c^high^MHCII^high^, CD11b^high^MHCII^high^CD11c^−^, GR-1^high^SSC^high^FSC^high^CD11c^−^ and CD19^+^CD3^−^CD11c^−^ cells, respectively. Cells were >98% pure and used for further analysis.

### Quantitative RT-PCR

Dendritic cells were stained with specific mAb and sorted from the LN of infected mice. Total RNA was extracted from dendritic cells using Tri reagent (Applied Biosystems, Carlsbad, Calif) and mini-elute columns (Qiagen) according to the manufacturer's protocol. cDNA was synthesized with Transcriptor First Strand cDNA synthesis kit (Roche), and real-time PCR was performed by using Lightcycler FastStart DNA Master PLUS SYBR Green I reaction mix (Roche) on a Lightcycler 480 II (Roche). Primers for IL-12p40: forward 5′ CTGGCCAGTACACCTGCCAC 3′ and reverse 5′ GTGCTTCCAACGCCAGTTC 3′, IL-18: forward 5′ TGGTTCCATGCTTTCTGG 3′ and reverse 5′ TCCGTATTACTGCGGTTGT 3′, IL-10: forward 5′ AGCCGGGAAGACAATAACTG 3′ and reverse 5′ CATTTCCGATAAGGCTTGG 3′, IL-23p19: forward 5′ CAGCTTAAGGATGCCCAGGTT 3′ and reverse 5′ TCTCACAGTTTCTCGATGCCA 3′ and βA subunit (Activin A): 5′ GAGAGGAGTGAACTGTTGCT 3′ and reverse 5′ TACAGCATGGACATGGGTCT 3′. Values were normalized according to the expression of the housekeeping genes *HPRT* or *rS12*.

### Nitric oxide synthase and arginase

Lymph node and footpad cells collected at week 4 after infection were restimulated with LPS (Sigma-Aldrich; 10 ng/ml). Supernatants were collected at 48 hours for quantification of nitric oxide [81] while arginase activity was measured in cell lysates [81]. Expression of intracellular iNOS and arginase was analyzed in CD11b^high^MHCII^high^CD11c^−^ macrophages and CD11c^high^MHCII^high^ DCs by flow cytometry using rabbit anti-mouse iNOS (Abcam) with goat anti-rabbit PE (Abcam) and goat anti-mouse arginase (Santa Cruz Biotechnology) with donkey anti-goat PE (Abcam). Purified goat IgG and rabbit IgG were used as controls.

### Statistics

Data is given as mean ± SEM. Statistical analysis was performed using the unpaired Student's *t* test or 1-way Anova with Bonferroni's post test, defining differences to IL-4Rα^-/lox^ mice as significant (*, p≤0.05; **, *p*≤0.01; ***, p≤0.001) unless otherwise stated. (Prism software: http://www.prism-software.com).

## Supporting Information

Figure S1 **CD11c^cre^IL-4Rα^-/lox^ mice are hypersusceptible to a 10-fold lower dose infection with** ***L. major*** **.** Mice were infected with *L. major* LV39 (MRHO/SV/59/P) parasite strain. Footpad swelling was measured at weekly intervals in mice (5 per group) infected subcutaneously with a 10-fold lower dose of 2×10^5^ stationary phase metacyclic *L. major* promastigotes into the hind footpad (A). “N” indicates necrosis or ulceration/mouse. Parasite burden was determined by limiting dilution of single-cell suspensions from homogenized footpads (B) and lymph nodes (C) at week 8 after infection. (D–F) Antigen-specific IgG2a (D), IgG1 (E) and total IgE (F) antibody production was quantified in sera by ELISA at week 8 post infection. (G–H) Total cells from the draining lymph node were incubated for 72 hrs with medium, αCD3 or soluble *Leishmania* antigen (SLA). The production of IFN-γ (G) and IL-4 (H) was determined by ELISA. A representative of two individual experiments is shown with mean values ±SEM. Statistical analysis was performed defining differences to IL-4Rα^-/lox^ mice as significant (*, *p*≤0.05, **, *p*≤0.01; ***, *p*≤0.001).
(TIF)

Figure S2 **Infiltration of GFP^+^-** ***L. major*** **parasites in immune cell populations in spleen during infection in CD11c^cre^IL-4Rα^-/lox^ mice.** Mice were infected subcutaneously with 2×10^6^ stationary phase metacyclic GFP-expressing *L. major* IL81 (MRHO/SV/59/P) strain into the hind footpad. At Day 0, Day 1, Day 3 and Week 4 after infection, total spleen cells were surface stained for dendritic cells (DCs-CD11c^high^MHCII^high^), Macrophages (Mph-CD11b^high^MHCII^high^CD11c^−^) and neutrophils (Neut-GR1^high^CD11c^−^). The percentage of infiltrating GFP^+^-infected cells were determined by flow cytometry.
(TIF)

Figure S3 **Viability of GFP^+^-** ***L. major*** **in immune cell populations during acute** ***L. major*** **IL81 infection by limiting dilution assay.** Experimental mice were infected subcutaneously with 2×10^6^ stationary phase metacyclic GFP-expressing *L. major* IL81 promastigotes into the hind footpad. At week 4 after infection, total lymph node cells were isolated and DCs (CD11c^high^MHCII^high^), macrophages (CD11b^high^MHCII^high^CD11c^−^), neutrophils (GR1^high^CD11c^−^) and B cells (CD19^+^CD3^−^CD11c^−^) were isolated by cell sorting on a FACS Vantage machine. Sorted cells were plated to determine viable parasite burden by limiting dilution assay in two-fold dilutions.
(TIF)

Figure S4 **Intracellular IL-12 and IL-10 in lymph node DCs.** Experimental mice were infected subcutaneously with 2×10^6^ stationary phase metacyclic *L. major* IL81 promastigotes into the hind footpad. Total lymph node cells were incubated with PMA/Ionomycin/Monensin for 4 h at 37°C, then surface-stained for CD11c^high^MHCII^high^ DCs followed by intracellular FACS staining for IL-12 and IL-10. Dot plots of percent cytokine producing cells are shown.
(TIF)

## References

[1] Murray HW, Berman JD, Davies CR, Saravia NG (2005) Advances in leishmaniasis. Lancet 366: 1561–77. 16257344 10.1016/S0140-6736(05)67629-5

[2] Reiner SL, Locksley RM (1995) The regulation of immunity to Leishmania major. Annu Rev Immunol 13: 151–77. 7612219 10.1146/annurev.iy.13.040195.001055

[3] Sacks D, Noben-Trauth N (2002) The immunology of susceptibility and resistance to Leishmania major in mice. Nat Rev Immunol 2: 845–58. 12415308 10.1038/nri933

[4] Alexander J, Satoskar AR, Russell DG (1999) Leishmania species: models of intracellular parasitism. J Cell Sci 112 Pt 18: 2993–3002. 10462516 10.1242/jcs.112.18.2993

[5] Locksley RM, Scott P (1991) Helper T-cell subsets in mouse leishmaniasis: induction, expansion and effector function. Immunol Today 12: A58–61. 1829891 10.1016/S0167-5699(05)80017-9

[6] Matthews DJ, Emson CL, McKenzie GJ, Jolin HE, Blackwell JM, et al (2000) IL-13 is a susceptibility factor for Leishmania major infection. J Immunol 164: 1458–62. 10640762 10.4049/jimmunol.164.3.1458

[7] Arendse B, Van Snick J, Brombacher F (2005) IL-9 is a susceptibility factor in Leishmania major infection by promoting detrimental Th2/type 2 responses. J Immunol 174: 2205–11. 15699153 10.4049/jimmunol.174.4.2205

[8] Kopf M, Brombacher F, Kohler G, Kienzle G, Widmann KH, et al (1996) IL-4-deficient Balb/c mice resist infection with Leishmania major. J Exp Med 184: 1127–36. 9064329 10.1084/jem.184.3.1127 PMC2192785

[9] Holscher C, Arendse B, Schwegmann A, Myburgh E, Brombacher F (2006) Impairment of alternative macrophage activation delays cutaneous leishmaniasis in nonhealing BALB/c mice. J Immunol 176: 1115–21. 16394000 10.4049/jimmunol.176.2.1115

[10] Iniesta V, Gomez-Nieto LC, Molano I, Mohedano A, Carcelen J, et al (2002) Arginase I induction in macrophages, triggered by Th2-type cytokines, supports the growth of intracellular Leishmania parasites. Parasite Immunol 24: 113–8. 11982856 10.1046/j.1365-3024.2002.00444.x

[11] Heinzel FP, Schoenhaut DS, Rerko RM, Rosser LE, Gately MK (1993) Recombinant interleukin 12 cures mice infected with Leishmania major. J Exp Med 177: 1505–9. 8097524 10.1084/jem.177.5.1505 PMC2191017

[12] Guler ML, Gorham JD, Hsieh CS, Mackey AJ, Steen RG, et al (1996) Genetic susceptibility to Leishmania: IL-12 responsiveness in TH1 cell development. Science 271: 984–7. 8584935 10.1126/science.271.5251.984

[13] Park AY, Hondowicz B, Kopf M, Scott P (2002) The role of IL-12 in maintaining resistance to Leishmania major. J Immunol 168: 5771–7. 12023378 10.4049/jimmunol.168.11.5771

[14] Stenger S, Thuring H, Rollinghoff M, Bogdan C (1994) Tissue expression of inducible nitric oxide synthase is closely associated with resistance to Leishmania major. J Exp Med 180: 783–93. 7520472 10.1084/jem.180.3.783 PMC2191630

[15] Mohrs M, Ledermann B, Kohler G, Dorfmuller A, Gessner A, et al (1999) Differences between IL-4− and IL-4 receptor alpha-deficient mice in chronic leishmaniasis reveal a protective role for IL-13 receptor signaling. J Immunol 162: 7302–8. 10358179

[16] Noben-Trauth N, Paul WE, Sacks DL (1999) IL-4− and IL-4 receptor-deficient BALB/c mice reveal differences in susceptibility to Leishmania major parasite substrains. J Immunol 162: 6132–40. 10229856

[17] Morris L, Troutt AB, Handman E, Kelso A (1992) Changes in the precursor frequencies of IL-4 and IFN-gamma secreting CD4+ cells correlate with resolution of lesions in murine cutaneous leishmaniasis. J Immunol 149: 2715–21. 1357029

[18] Belkaid Y, Mendez S, Lira R, Kadambi N, Milon G, et al (2000) A natural model of Leishmania major infection reveals a prolonged “silent” phase of parasite amplification in the skin before the onset of lesion formation and immunity. J Immunol 165: 969–77. 10878373 10.4049/jimmunol.165.2.969

[19] Himmelrich H, Launois P, Maillard I, Biedermann T, Tacchini-Cottier F, et al (2000) In BALB/c mice, IL-4 production during the initial phase of infection with Leishmania major is necessary and sufficient to instruct Th2 cell development resulting in progressive disease. J Immunol 164: 4819–25. 10779790 10.4049/jimmunol.164.9.4819

[20] Hochrein H, O'Keeffe M, Luft T, Vandenabeele S, Grumont RJ, et al (2000) Interleukin (IL)-4 is a major regulatory cytokine governing bioactive IL-12 production by mouse and human dendritic cells. J Exp Med 192: 823–33. 10993913 10.1084/jem.192.6.823 PMC2193283

[21] Biedermann T, Zimmermann S, Himmelrich H, Gumy A, Egeter O, et al (2001) IL-4 instructs TH1 responses and resistance to Leishmania major in susceptible BALB/c mice. Nat Immunol 2: 1054–60. 11600887 10.1038/ni725

[22] Lutz MB, Schnare M, Menges M, Rossner S, Rollinghoff M, et al (2002) Differential functions of IL-4 receptor types I and II for dendritic cell maturation and IL-12 production and their dependency on GM-CSF. J Immunol 169: 3574–80. 12244147 10.4049/jimmunol.169.7.3574

[23] Yao Y, Li W, Kaplan MH, Chang CH (2005) Interleukin (IL)-4 inhibits IL-10 to promote IL-12 production by dendritic cells. J Exp Med 201: 1899–903. 15967820 10.1084/jem.20050324 PMC2212025

[24] Radwanska M, Cutler AJ, Hoving JC, Magez S, Holscher C, et al (2007) Deletion of IL-4Ralpha on CD4 T cells renders BALB/c mice resistant to Leishmania major infection. PLoS Pathog 3: e68. 17500591 10.1371/journal.ppat.0030068 PMC1867380

[25] Banchereau J, Briere F, Caux C, Davoust J, Lebecque S, et al (2000) Immunobiology of dendritic cells. Annu Rev Immunol 18: 767–811. 10837075 10.1146/annurev.immunol.18.1.767

[26] Brandonisio O, Spinelli R, Pepe M (2004) Dendritic cells in Leishmania infection. Microbes Infect 6: 1402–9. 15596127 10.1016/j.micinf.2004.10.004

[27] Caton ML, Smith-Raska MR, Reizis B (2007) Notch-RBP-J signaling controls the homeostasis of CD8− dendritic cells in the spleen. J Exp Med 204: 1653–64. 17591855 10.1084/jem.20062648 PMC2118632

[28] Herbert DR, Holscher C, Mohrs M, Arendse B, Schwegmann A, et al (2004) Alternative macrophage activation is essential for survival during schistosomiasis and downmodulates T helper 1 responses and immunopathology. Immunity 20: 623–35. 15142530 10.1016/s1074-7613(04)00107-4

[29] Cook PC, Jones LH, Jenkins SJ, Wynn TA, Allen JE, et al (2012) Alternatively activated dendritic cells regulate CD4+ T-cell polarization in vitro and in vivo. Proc Natl Acad Sci U S A 109: 9977–82. 22660926 10.1073/pnas.1121231109 PMC3382483

[30] Laskay T, Diefenbach A, Rollinghoff M, Solbach W (1995) Early parasite containment is decisive for resistance to Leishmania major infection. Eur J Immunol 25: 2220–7. 7664785 10.1002/eji.1830250816

[31] De Trez C, Magez S, Akira S, Ryffel B, Carlier Y, et al (2009) iNOS-producing inflammatory dendritic cells constitute the major infected cell type during the chronic Leishmania major infection phase of C57BL/6 resistant mice. PLoS Pathog 5: e1000494. 19557162 10.1371/journal.ppat.1000494 PMC2695779

[32] Lopez Kostka S, Dinges S, Griewank K, Iwakura Y, Udey MC, et al (2009) IL-17 promotes progression of cutaneous leishmaniasis in susceptible mice. J Immunol 182: 3039–46. 19234200 10.4049/jimmunol.0713598 PMC2658650

[33] Ogawa K, Funaba M, Chen Y, Tsujimoto M (2006) Activin A functions as a Th2 cytokine in the promotion of the alternative activation of macrophages. J Immunol 177: 6787–94. 17082592 10.4049/jimmunol.177.10.6787

[34] Sadick MD, Heinzel FP, Holaday BJ, Pu RT, Dawkins RS, et al (1990) Cure of murine leishmaniasis with anti-interleukin 4 monoclonal antibody. Evidence for a T cell-dependent, interferon gamma-independent mechanism. J Exp Med 171: 115–27. 2104918 10.1084/jem.171.1.115 PMC2187647

[35] Launois P, Maillard I, Pingel S, Swihart KG, Xenarios I, et al (1997) IL-4 rapidly produced by V beta 4 V alpha 8 CD4+ T cells instructs Th2 development and susceptibility to Leishmania major in BALB/c mice. Immunity 6: 541–9. 9175832 10.1016/s1074-7613(00)80342-8

[36] Erb KJ, Ruger B, von Brevern M, Ryffel B, Schimpl A, et al (1997) Constitutive expression of interleukin (IL)-4 in vivo causes autoimmune-type disorders in mice. J Exp Med 185: 329–39. 9016881 10.1084/jem.185.2.329 PMC2196114

[37] Noben-Trauth N, Kropf P, Muller I (1996) Susceptibility to Leishmania major infection in interleukin-4-deficient mice. Science 271: 987–90. 8584936 10.1126/science.271.5251.987

[38] Mencacci A, Del Sero G, Cenci E, d'Ostiani CF, Bacci A, et al (1998) Endogenous interleukin 4 is required for development of protective CD4+ T helper type 1 cell responses to Candida albicans. J Exp Med 187: 307–17. 9449711 10.1084/jem.187.3.307 PMC2212115

[39] Schuler T, Qin Z, Ibe S, Noben-Trauth N, Blankenstein T (1999) T helper cell type 1-associated and cytotoxic T lymphocyte-mediated tumor immunity is impaired in interleukin 4-deficient mice. J Exp Med 189: 803–10. 10049944 10.1084/jem.189.5.803 PMC2192943

[40] Bagley J, Sawada T, Wu Y, Iacomini J (2000) A critical role for interleukin 4 in activating alloreactive CD4 T cells. Nat Immunol 1: 257–61. 10973285 10.1038/79811

[41] Salerno A, Dieli F, Sireci G, Bellavia A, Asherson GL (1995) Interleukin-4 is a critical cytokine in contact sensitivity. Immunology 84: 404–9. 7751023 PMC1415118

[42] Padilla J, Daley E, Chow A, Robinson K, Parthasarathi K, et al (2005) IL-13 regulates the immune response to inhaled antigens. J Immunol 174: 8097–105. 15944318 10.4049/jimmunol.174.12.8097

[43] Noben-Trauth N, Lira R, Nagase H, Paul WE, Sacks DL (2003) The relative contribution of IL-4 receptor signaling and IL-10 to susceptibility to Leishmania major. J Immunol 170: 5152–8. 12734362 10.4049/jimmunol.170.10.5152

[44] Anderson CF, Mendez S, Sacks DL (2005) Nonhealing infection despite Th1 polarization produced by a strain of Leishmania major in C57BL/6 mice. J Immunol 174: 2934–41. 15728505 10.4049/jimmunol.174.5.2934

[45] Diefenbach A, Schindler H, Rollinghoff M, Yokoyama WM, Bogdan C (1999) Requirement for type 2 NO synthase for IL-12 signaling in innate immunity. Science 284: 951–5. 10320373 10.1126/science.284.5416.951

[46] Liew FY, Millott S, Parkinson C, Palmer RM, Moncada S (1990) Macrophage killing of Leishmania parasite in vivo is mediated by nitric oxide from L-arginine. J Immunol 144: 4794–7. 2351828

[47] Stenger S, Donhauser N, Thuring H, Rollinghoff M, Bogdan C (1996) Reactivation of latent leishmaniasis by inhibition of inducible nitric oxide synthase. J Exp Med 183: 1501–14. 8666908 10.1084/jem.183.4.1501 PMC2192515

[48] Kane MM, Mosser DM (2001) The role of IL-10 in promoting disease progression in leishmaniasis. J Immunol 166: 1141–7. 11145695 10.4049/jimmunol.166.2.1141

[49] Gazzinelli RT, Oswald IP, James SL, Sher A (1992) IL-10 inhibits parasite killing and nitrogen oxide production by IFN-gamma-activated macrophages. J Immunol 148: 1792–6. 1541819

[50] Bogdan C, Vodovotz Y, Nathan C (1991) Macrophage deactivation by interleukin 10. J Exp Med 174: 1549–55. 1744584 10.1084/jem.174.6.1549 PMC2119047

[51] Dewals BG, Marillier RG, Hoving JC, Leeto M, Schwegmann A, et al (2010) IL-4Ralpha-independent expression of mannose receptor and Ym1 by macrophages depends on their IL-10 responsiveness. PLoS Negl Trop Dis 4: e689. 20502521 10.1371/journal.pntd.0000689 PMC2872644

[52] Roberts SC, Tancer MJ, Polinsky MR, Gibson KM, Heby O, et al (2004) Arginase plays a pivotal role in polyamine precursor metabolism in Leishmania. Characterization of gene deletion mutants. J Biol Chem 279: 23668–78. 15023992 10.1074/jbc.M402042200

[53] Brombacher F (2000) The role of interleukin-13 in infectious diseases and allergy. Bioessays 22: 646–56. 10878577 10.1002/1521-1878(200007)22:7<646::AID-BIES7>3.0.CO;2-9

[54] Colotti G, Ilari A (2011) Polyamine metabolism in Leishmania: from arginine to trypanothione. Amino Acids 40: 269–85. 20512387 10.1007/s00726-010-0630-3

[55] Kropf P, Fuentes JM, Fahnrich E, Arpa L, Herath S, et al (2005) Arginase and polyamine synthesis are key factors in the regulation of experimental leishmaniasis in vivo. FASEB J 19: 1000–2. 15811879 10.1096/fj.04-3416fje

[56] Kropf P, Freudenberg MA, Modolell M, Price HP, Herath S, et al (2004) Toll-like receptor 4 contributes to efficient control of infection with the protozoan parasite Leishmania major. Infect Immun 72: 1920–8. 15039311 10.1128/IAI.72.4.1920-1928.2004 PMC375159

[57] Serbina NV, Salazar-Mather TP, Biron CA, Kuziel WA, Pamer EG (2003) TNF/iNOS-producing dendritic cells mediate innate immune defense against bacterial infection. Immunity 19: 59–70. 12871639 10.1016/s1074-7613(03)00171-7

[58] Copin R, De Baetselier P, Carlier Y, Letesson JJ, Muraille E (2007) MyD88-dependent activation of B220-CD11b+LY-6C+ dendritic cells during Brucella melitensis infection. J Immunol 178: 5182–91. 17404301 10.4049/jimmunol.178.8.5182

[59] Schilling S, Glaichenhaus N (2001) T cells that react to the immunodominant Leishmania major LACK antigen prevent early dissemination of the parasite in susceptible BALB/c mice. Infect Immun 69: 1212–4. 11160025 10.1128/IAI.69.2.1212-1214.2001 PMC98009

[60] Guy RA, Belosevic M (1995) Response of scid mice to establishment of Leishmania major infection. Clin Exp Immunol 100: 440–5. 7774053 10.1111/j.1365-2249.1995.tb03719.x PMC1534477

[61] Kautz-Neu K, Kostka SL, Dinges S, Iwakura Y, Udey MC, et al (2011) A role for leukocyte-derived IL-1RA in DC homeostasis revealed by increased susceptibility of IL-1RA-deficient mice to cutaneous leishmaniasis. J Invest Dermatol 131: 1650–9. 21525884 10.1038/jid.2011.99 PMC6999703

[62] Murray HW, Lu CM, Mauze S, Freeman S, Moreira AL, et al (2002) Interleukin-10 (IL-10) in experimental visceral leishmaniasis and IL-10 receptor blockade as immunotherapy. Infect Immun 70: 6284–93. 12379707 10.1128/IAI.70.11.6284-6293.2002 PMC130311

[63] Marzieh Amini HN, Mahin Farahmand (2008) Pathogenicity Variations of Susceptibility and Resistance to Leishmania major MRHO/IR/75/ER Strain in BALB/c and C57BL/6 mice. Iranian J Parasitol 3: 51–59.

[64] Nieto CG, Vinuelas J, Blanco A, Garcia-Alonso M, Verdugo SG, et al (1996) Detection of Leishmania infantum amastigotes in canine choroid plexus. Vet Rec 139: 346–7. 8903015 10.1136/vr.139.14.346

[65] Abreu-Silva AL, Calabrese KS, Tedesco RC, Mortara RA, da Costa Goncalves SC (2003) Central nervous system involvement in experimental infection with Leishmania (Leishmania) amazonensis. Am J Trop Med Hyg 68: 661–5. 12887024

[66] Vinuelas J, Garcia-Alonso M, Ferrando L, Navarrete I, Molano I, et al (2001) Meningeal leishmaniosis induced by Leishmania infantum in naturally infected dogs. Vet Parasitol 101: 23–7. 11587830 10.1016/s0304-4017(01)00413-7

[67] Petersen CA, Greenlee MH (2011) Neurologic Manifestations of Leishmania spp. Infection. J Neuroparasitology 2: N110401. 21666756 PMC3110707

[68] Drevets DA, Leenen PJ (2000) Leukocyte-facilitated entry of intracellular pathogens into the central nervous system. Microbes Infect 2: 1609–18. 11113380 10.1016/s1286-4579(00)01317-4

[69] Drevets DA, Leenen PJ, Greenfield RA (2004) Invasion of the central nervous system by intracellular bacteria. Clin Microbiol Rev 17: 323–47. 15084504 10.1128/CMR.17.2.323-347.2004 PMC387409

[70] Muraille E, De Trez C, Pajak B, Torrentera FA, De Baetselier P, et al (2003) Amastigote load and cell surface phenotype of infected cells from lesions and lymph nodes of susceptible and resistant mice infected with Leishmania major. Infect Immun 71: 2704–15. 12704145 10.1128/IAI.71.5.2704-2715.2003 PMC153240

[71] Schreiber HA, Harding JS, Hunt O, Altamirano CJ, Hulseberg PD, et al (2011) Inflammatory dendritic cells migrate in and out of transplanted chronic mycobacterial granulomas in mice. J Clin Invest 121: 3902–13. 21911937 10.1172/JCI45113 PMC3195456

[72] Drevets DA, Bronze MS (2008) Listeria monocytogenes: epidemiology, human disease, and mechanisms of brain invasion. FEMS Immunol Med Microbiol 53: 151–65. 18462388 10.1111/j.1574-695X.2008.00404.x

[73] Laskay T, van Zandbergen G, Solbach W (2003) Neutrophil granulocytes–Trojan horses for Leishmania major and other intracellular microbes? Trends Microbiol 11: 210–4. 12781523 10.1016/s0966-842x(03)00075-1

[74] Radu DL, Noben-Trauth N, Hu-Li J, Paul WE, Bona CA (2000) A targeted mutation in the IL-4Ralpha gene protects mice against autoimmune diabetes. Proc Natl Acad Sci U S A 97: 12700–4. 11050183 10.1073/pnas.230431397 PMC18827

[75] Tepper RI, Pattengale PK, Leder P (1989) Murine interleukin-4 displays potent anti-tumor activity in vivo. Cell 57: 503–12. 2785856 10.1016/0092-8674(89)90925-2

[76] Golumbek PT, Lazenby AJ, Levitsky HI, Jaffee LM, Karasuyama H, et al (1991) Treatment of established renal cancer by tumor cells engineered to secrete interleukin-4. Science 254: 713–6. 1948050 10.1126/science.1948050

[77] Traidl C, Jugert F, Krieg T, Merk H, Hunzelmann N (1999) Inhibition of allergic contact dermatitis to DNCB but not to oxazolone in interleukin-4-deficient mice. J Invest Dermatol 112: 476–82. 10201532 10.1046/j.1523-1747.1999.00550.x

[78] D'Andrea A, Ma X, Aste-Amezaga M, Paganin C, Trinchieri G (1995) Stimulatory and inhibitory effects of interleukin (IL)-4 and IL-13 on the production of cytokines by human peripheral blood mononuclear cells: priming for IL-12 and tumor necrosis factor alpha production. J Exp Med 181: 537–46. 7836910 10.1084/jem.181.2.537 PMC2191875

[79] Masic A, Hurdayal R, Nieuwenhuizen NE, Brombacher F, Moll H (2012) Dendritic Cell-Mediated Vaccination Relies on Interleukin-4 Receptor Signaling to Avoid Tissue Damage after Leishmania major Infection of BALB/c Mice. PLoS Negl Trop Dis 6: e1721. 22802978 10.1371/journal.pntd.0001721 PMC3389028

[80] Lutz MB, Kukutsch N, Ogilvie AL, Rossner S, Koch F, et al (1999) An advanced culture method for generating large quantities of highly pure dendritic cells from mouse bone marrow. J Immunol Methods 223: 77–92. 10037236 10.1016/s0022-1759(98)00204-x

[81] Modolell M, Corraliza IM, Link F, Soler G, Eichmann K (1995) Reciprocal regulation of the nitric oxide synthase/arginase balance in mouse bone marrow-derived macrophages by TH1 and TH2 cytokines. Eur J Immunol 25: 1101–4. 7537672 10.1002/eji.1830250436

