# Supplementary figures and images for: Deletion of IL-4 Receptor Alpha on Dendritic Cells Renders BALB/c Mice Hypersusceptible to Leishmania major Infection
Source: PLoS Pathog. 2013 Oct 24;9(10):e1003699. doi: 10.1371/journal.ppat.1003699 (PMC3812013; doi:10.1371/journal.ppat.1003699)

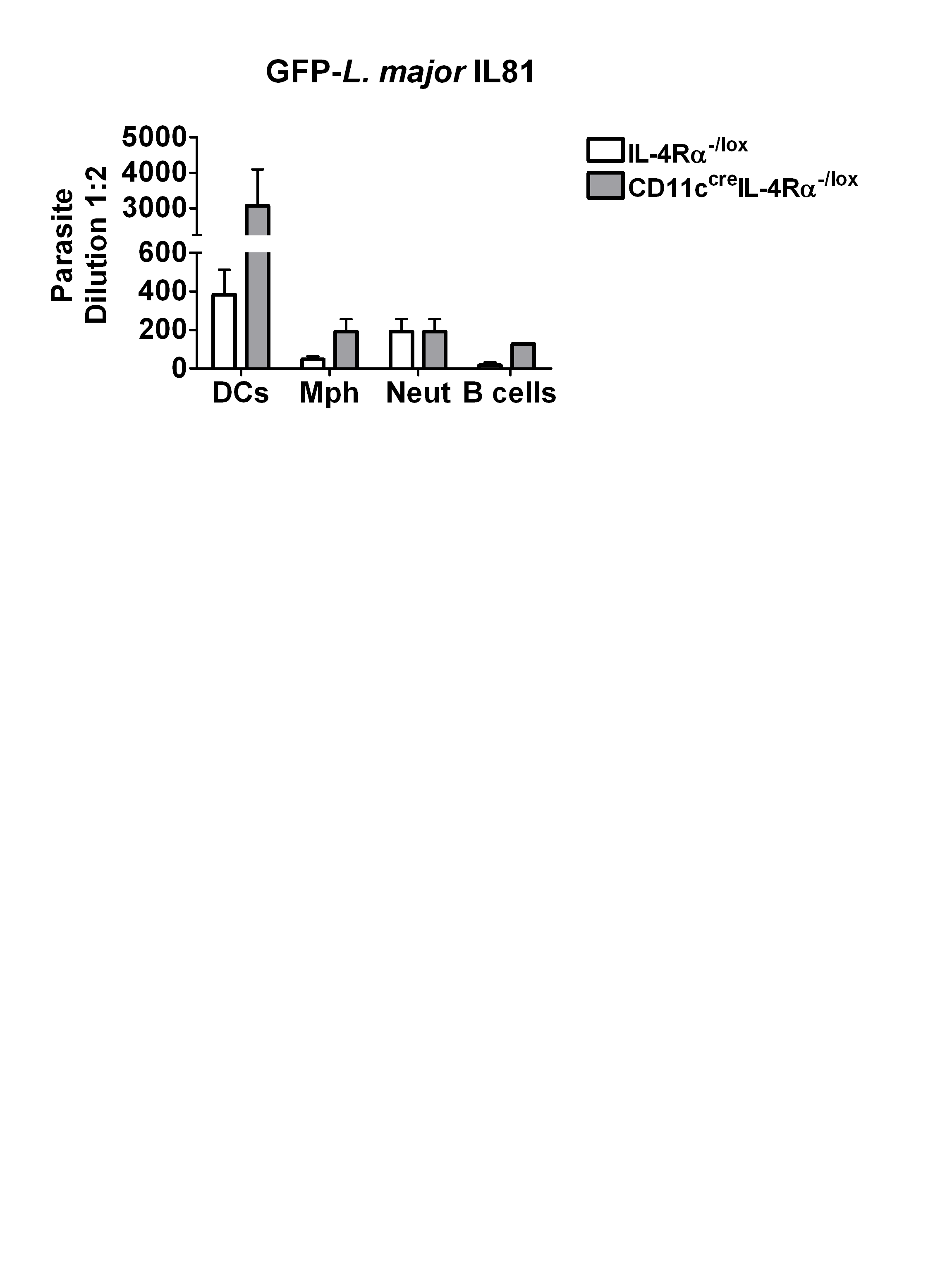

Supplement: Figure S3 — Viability of GFP+- L. major in immune cell populations during acute L. major IL81 infection by limiting dilution assay. Experimental mice were infected subcutaneously with 2×10^6^ stationary phase metacyclic GFP-expressing L. major IL81 promastigotes into the hind footpad. At week 4 after infection, total lymph node cells were isolated and DCs (CD11chighMHCIIhigh), macrophages (CD11bhighMHCIIhighCD11c−), neutrophils (GR1highCD11c−) and B cells (CD19+CD3−CD11c−) were isolated by cell sorting on a FACS Vantage machine. Sorted cells were plated to determine viable parasite burden by limiting dilution assay in two-fold dilutions. (TIF) [file ppat.1003699.s003.tif]
